# PBX3/MEK/ERK1/2/LIN28/let-7b positive feedback loop enhances mesenchymal phenotype to promote glioblastoma migration and invasion

**DOI:** 10.1186/s13046-018-0841-0

**Published:** 2018-07-17

**Authors:** Xiupeng Xu, Zhongyuan Bao, Yinlong Liu, Kuan Jiang, Tongle Zhi, Dong Wang, Liang Fan, Ning Liu, Jing Ji

**Affiliations:** 1 Department of Neurosurgery, the First Affiliated Hospital of Nanjing Medical University, Guangzhou Road 300, Nanjing, Jiangsu China; 2 Department of Neurosurgery, Suzhou Municipal Hospital, Suzhou, Jiangsu China; 3 Department of Neurosurgery, Yixing People’s Hospital, Yixing, Jiangsu China

**Keywords:** PBX3, Mesenchymal transition, ERK1/2, LIN28/let-7b, C-myc, GBM

## Abstract

**Background:**

Brain invasion by glioblastoma (GBM) determines recurrence and prognosis in patients, which is, in part, attributed to increased mesenchymal transition. Here, we report evidence favoring such a role for the Pre-B-cell leukemia homebox (PBX) family member PBX3.

**Methods:**

Western blot, immunohistochemistry, qRT-PCR and datasets mining were used to determined proteins or genes expression levels. Wound-healing and transwell assays were used to examine the invasive abilities of GBM cells. Dual-luciferase reporter assays were used to determine how let-7b regulates PBX3. Chromatin-immunoprecipitation (ChIP) and rescue experiments were performed to investigate the involved molecular mechanisms. Orthotopic mouse models were used to assess the role of PBX3 in vivo.

**Results:**

We found that PBX3 expression levels positively correlated with glioma mesenchymal markers. Ectopic expression of PBX3 promoted invasive phenotypes and triggered the expression of mesenchymal markers, whereas depletion of PBX3 reduced GBM cell invasive abilities and decreased the expression of mesenchymal markers. In addition, inhibition of PBX3 attenuated transforming growth factor-β (TGFβ)-induced GBM mesenchymal transition. Mechanistic studies revealed that PBX3 mediated GBM mesenchymal transition through activation of MEK/ERK1/2, leading to increased expression of LIN28 by c-myc. Increased LIN28 inhibited let-7b biogenesis, which then promoted the pro-invasive genes, such as HMGA2 and IL-6. Furthermore, let-7b suppressed PBX3 by directly targeting 3′-UTR of PBX3. Thus, repressed let-7b by PBX3 amplifies PBX3 signaling and forms a positive feedback loop to promote GBM mesenchymal transition.

**Conclusions:**

These data highlight the importance of PBX3 as a key driver of mesenchymal transition and potential therapeutic target.

**Electronic supplementary material:**

The online version of this article (10.1186/s13046-018-0841-0) contains supplementary material, which is available to authorized users.

## Background

Glioblastoma (GBM) is the most common and lethal malignant brain tumor in adults [1]. Despite recent advances in therapeutic strategies, including surgical resection followed by radiotherapy and temozolomide chemotherapy, the average survival of patients with GBM is approximately 14 months after the initial diagnosis [2, 3]. This dismal prognosis may be partly attributed to the highly invasive nature of GBM, which favors its infiltration into the surrounding normal brain parenchymal, making total surgical resection impossible [4, 5]. Thus, there is an urgent need to identify therapies that decrease GBM invasion and increase patient survival.

The inability to define different patient outcomes based on the traditional histopathological classification suggests a large problem in our understanding of the classification of GBM [6]. To better understand determinants of GBM malignant progression, genomic- and genetic-based molecular stratification of GBM were performed [7]. Although inconsistence were observed among different classification studies, the proneural and mesenchymal subtypes were highly consistent in literatures [8–11]. The proneural subtype is characterized by the high expression of genes involved in neurogenesis, and is associated with a favorable outcome [10]. By contrast, the mesenchymal subtype shows high expression of genes correlated with invasion, motility and stemness, and is associated with poor clinical outcomes [12, 13]. Importantly, proneural-to-mesenchymal transition (PMT) was frequently observed upon GBM relapse [14, 15]. Subsequent studies demonstrated that this process is mediated by several key transcriptional factors, such as nuclear factor κB (NF-κB) [16], Snail Family Transcriptional Repressor 1 (SNAI1) [17] and Signal Transducer and Activator of Transcription 3 (STAT3) [15]. However, detailed molecular mechanisms that promote PMT remain ambiguous. Given the malignant behaviors of mesenchymal GBM, uncovering the underlying molecular mechanisms that responsible for maintaining mesenchymal phenotype is urgently needed for targeted therapies.

Pre-B-cell leukemia homebox 3 (PBX3) was first identified in 1991 by Monic et al. [18], which belongs to the three-amino acid-loop-extension family of homeodomain transcription factors. Subsequent studies showed that PBX3 is overexpressed in various human cancers, including prostate cancer [19], colorectal cancer [20], gastric cancer [21], hepatocellular carcinoma [22], and multiple myeloma [23]. Functional studies revealed that upregulated PBX3 promotes cancer cell malignant behaviors, such as proliferation, migration, invasion, cell cycle progression, drug resistance and cancer stemness [21–24]. Moreover, survival analysis demonstrated that PBX3 expression is predictive of poor prognosis in some malignancies [20, 22, 25]. Our group recently reported that PBX3 is overexpressed in GBM and promotes GBM migration, invasion, proliferation and cell cycle progression [26, 27]. As PMT is associated with malignant phenotypes of GBM, we hypothesized that upregulated PBX3 might be involved in promoting PMT in GBM.

Aberrant expression of LIN28/let-7 axis has been well documented in various malignancies, including GBM [28]. Experimentally, LIN28/let-7 axis has been shown to induce cancer cell invasive phenotypes and mensenchymal transition by regulating various let-7 targets, such as high mobility group A2 (HMGA2) [29, 30]. Importantly, LIN28/let-7 axis represents a critical downstream target of MEK/ERK1/2 pathway and mediates ERK1/2-driven mesenchymal transition [30]. As PBX3 activates MEK/ERK1/2 pathway to promote malignant phenotypes in some cancers [25], we hypothesized that PBX3 may promote GBM mesenchymal phenotype through MEK/ERK1/2 pathway mediated LIN28/let-7 axis activation.

Here, we characterized the role of PBX3 in regulating PMT process. Our results demonstrated that upregulation/downregulation of PBX3 increases/decreases mesenchymal phenotype of GBM. Mechanically, we showed that PBX3 enhances mesenchymal phenotype of GBM through a positive feedback loop involving activation of MEK, ERK1/2, c-myc, and LIN28, leading to inhibition of the let-7b expression and upregulation of its targets. Finally, we found that PBX3 is required for TGF-β-induced GBM mesenchymal transition.

## Methods

### Database mining and human tissue samples

Glioma gene expression data were downloaded from three datasets: Repository for Molecular Brain Neoplasia Data (Rembrandt; http://caintegratorinfo.nci.nih.gov/rembrandt); and the National Center for Biotechnology Information Gene Expression Omnibus (NCBI-GEO) datasets GSE4290 and GSE59612 (http://www.ncbi.nlm.gov.geo/; accession nos. GSE4290 and GSE59612). Forty-five GBM samples were collected from the Department of Neurosurgery, The First Affiliated Hospital of Nanjing Medical University between 2011 and 2014. Informed consent for the use of samples was obtained from all patients. Our study was approved by the institutional review board and ethics committee of Nanjing Medical University.

#### Gene set enrichment analysis (GSEA)

The GSEA was performed using software downloaded from the Broad Institute (http://www.broadinstitute.org/gsea/index.jsp) with H (hallmark gene sets) collection. We divided the GBM cohort in Rembrandt into two groups with high PBX3 expression or low PBX3 expression. The Gene Cluster Text file (.gct) was generated from the Rembrandt dataset. The Categorical Class file (.cls) was prepared based on the PBX3 mRNA levels of GBM patients in the Rembrandt dataset. Using a permutation test at 1000 times, we finally identified gene signatures that were enriched in the PBX3 high expression group.

### Cell culture, transfection and drug treatment

U87 and U251 cells were purchased from the American Type Culture Collection (ATCC, Manassas, VA). Cells were cultured in Dulbecco’s modified Eagle’s medium (DMEM; Hyclone, USA) supplemented with 10% fetal bovine serum (Gibco, Invitrogen, Carlsbad, CA, USA) at 37 °C in a humidified atmosphere with 5% CO_2_.

Lentiviruses carrying PBX3 or vectors or siRNA-PBX3 or siRNA-NC were purchased from Genepharma (Shanghai, China). Stable U87 and U251 cells were established by lentiviral infection and puromycin selection as manufacturer’s protocol. Although, our previous study showed that PBX3 protein levels in H4 and U118 cell lines were significantly lower than that in U87 and U251 cells [27], we did not select H4 and U118 cells for overexpression studies. The main reason is the low ovexpression efficiency of PBX3 in both H4 and U118 cells. Let-7b mimic and inhibitor were purchased from RiboBio (Guangzhou, China) and transfected into cells using Lipofectamine RNAiMAX Reagent (Life Technologies, Grand Island, NY) according to manufacturer’s protocol. siRNAs or plasmids were synthesized by Genepharma and transfected into cells using Lipofectamine 2000 Transfection Reagent (Invitrogen).

Recombinant human transforming growth factor-β (TGFβ), U0126 and phorbol 12-myristate 13-acetate (PMA) were purchased from Sigma, and diluted according to each manufacturer’s protocol. The dose of each drug was used as previously described [17, 31].

### RNA extraction and quantitative real-time polymerase chain reaction (qRT-PCR)

Total RNA extraction and qRT-PCR for mRNA were performed as previously described [26]. Primers used in this study were listed as follows: PBX3 forward 5′-CAAGTCGGAGCCAATGTG-3′ and reverse 5′-ATGTAGCTCAGGGAAAAGTG-3′; N-cadherin forward 5′-GAAGGAGGTGGGGAGGAAGATA-3′ and reverse 5′-GGTGGTCTCTGACGAGGTAAACA-3′; ZEB1 forward 5′-AGTTTACCTTCCAGCAGCCCTAC-3′; and reverse 5′-AGCTCTTCTGCACTTGGTTGTG-3′; Slug forward 5′-AGACCCCCATGCCATTGAAG-3′ and reverse 5′-GGCCAGCCCAGAAAAAGTTG-3′; CD44 forward 5′-CACAACAACACAAATGGCTG-3′ and reverse 5′-CAATGCCTGATCCAGAAAAA-3′; IL-6 forward 5′-TCCAGAACAGATTTGAGAGTAGTG-3′ and reverse 5′-GCATTTGTGGTTGGGTCAGG-3′; HMGA2 forward 5′-CACTTCAGCCCAGGGACAAC-3′ and reverse 5′-GCCTCTTGGGCGTTTTTCTC-3′; β-actin forward 5′-GTGATCTCCTTCTGCATCCTGT-3′ and reverse 5′-CCACGAAACTACCTTCAACTCC-3′; qRT-PCR for let-7b was performed using commercially available TaqMan® MicroRNA Assays (#4373168, Applied Biosystems, Darmstadt, Germany) according to manufacturer’s protocol. The specificity of PCR was confirmed by melting curves and PCR product sequencing.

### Western blot analysis

Protein extraction, quantification and immunoblotting were performed as our previously described [27]. The antibodies used in this study were: PBX3 (1:500, ab109173), N-cadherin (1:750, ab18203), ZEB1 (1:500, ab180905), Slug (1:750, ab180714), CD44 (1:750, ab51037), c-myc (1:500, ab32072), LIN28 (1:500, ab46020) and β-actin (1:2000, ab8226) all from Abcam (Cambridge, UK). HMGA2 (1:1000, no. 8179), STAT3 (1:1000, no. 9139), phospho-STAT3 (1:500, no. 9145), MEK1/2 (1:1500, no. 4694), phospho-MEK1/2 (1:1000, no. 4370), ERK1/2 (1:2000, no. 9194,) and phospho-ERK1/2 (1:1000, no. 4370) all from Cell Signaling Technologies (Danvers, MA, USA).

### In vitro cell migration and invasion assays

The invasive capabilities of glioma cells were determined as our previously reported [26].

### Immunohistochemsitry

Tissues were fixed in 4% paraformaldehyde for two 48 h and paraffin embedded in a regular way. Tissue sections (4 μm) were treated and stained with the following antibodies: PBX3 (1:75, ab109173), N-cadherin (1:100, ab18203), ZEB1 (1:150, ab180905), Slug (1:150, ab180714), CD44 (1:100, ab51037).

### Quantitative chromatin immunoprecipitation analysis

Chromatin-immunoprecipitation (ChIP) assays were carried out using a ChIP Assay Kit (#17408, Millipore) according to the manufacturer’s protocol. The chromatin fragments were immunoprecipitated with 2 μg of antibodies against either c-myc (ab32) or Jun (ab31419). The primer sequences used in this study were as follows: LIN28 (c-myc) forward 5′-GGGAGGGCCCATTCATTTC-3′ and reverse 5′-GGGTCCCCAAAGCAGATACA-3′; Wnt5a (c-myc) forward 5′-GTCGGGAAGTGGTCAAGGTT-3′ and reverse 5′-AAGTGCCAGAGACAGATGCT-3′; CyclinD1 (Jun) forward 5′-GTCCCAGGCAGAGGGGAC-3′ and reverse 5′-CGGCAATTTAACCGGGAGA-3′; β-globin (negative control) forward 5′-AGTGCCAGAGCCAAGGA-3′ and reverse 5′-CAGGGTGAGGTCTAAGTGATGACA-3′; and rRNA (internal control) forward 5′-ATTAGTCAGCGGAGGAAAAGAAAC-3′ and reverse 5′-TCGCCGTTACTGAGGGAATC-3′.

### Luciferase reporter assay

To determine whether let-7b directly binds to the PBX3 3′-UTR, dual luciferase reporter assays were conducted. Wild-type (WT) and mutated putative let-7b-binding sites were amplified and cloned into the XbaI site of a pGL3 control vector (Invitrogen). The following reporter assays were performed in a regular way as our previously described [26].

### Determination of interleukin-6 concentrations in supernatant of cultured cells

The collected supernatant was serially diluted, and levels of IL-6 of different groups were measured by enzyme-linked immunosorbent assay (ELISA; R&D Systems Inc., Minneapolis, MN).

### Orthotopic GBM model

Female Bagg albino (BALB)/c nude mice at 5 weeks were purchased from the Shanghai Experimental Animal Center of the Chinese Academy of Sciences and maintained in specific pathogen-free conditions for 1 week. To established intracranial GBMs, LV-siRNA-NC- or LV-siRNA-PBX3-transfected U87 cells (2.5 × 10^5^) were stereotactically injected (12 mice for each group). Three weeks later, all mice were killed by rapid decapitation and brains were extracted and stored in liquid nitrogen. All experimental procedures were in accordance with the Guide for the Care and Use of Laboratory Animals of the National Institutes of Health and approved by the Nanjing Medical University Animal Experimental Ethics Committee.

### Statistical analysis

Statistical analyses were performed using GraphPad Prism 5.0 (GraphPad Software, Inc., LaJolla, CA, USA). Data were presented as mean ± SD. The significance of differences between groups was tested by two-tailed Student’s *t*-test. Correlations between PBX3 and N-cadherin, ZEB1, Slug, CD44, let-7b, HMGA2, and IL-6 expressions were analyzed with Pearson’s correlation method. Values of *p* < 0.05 were considered statistically significant.

## Results

### PBX3 is upregulated in mesenchymal gliomas and promotes glioma migration and invasion via enhancing mesenchymal transition

**Fig. 1:**
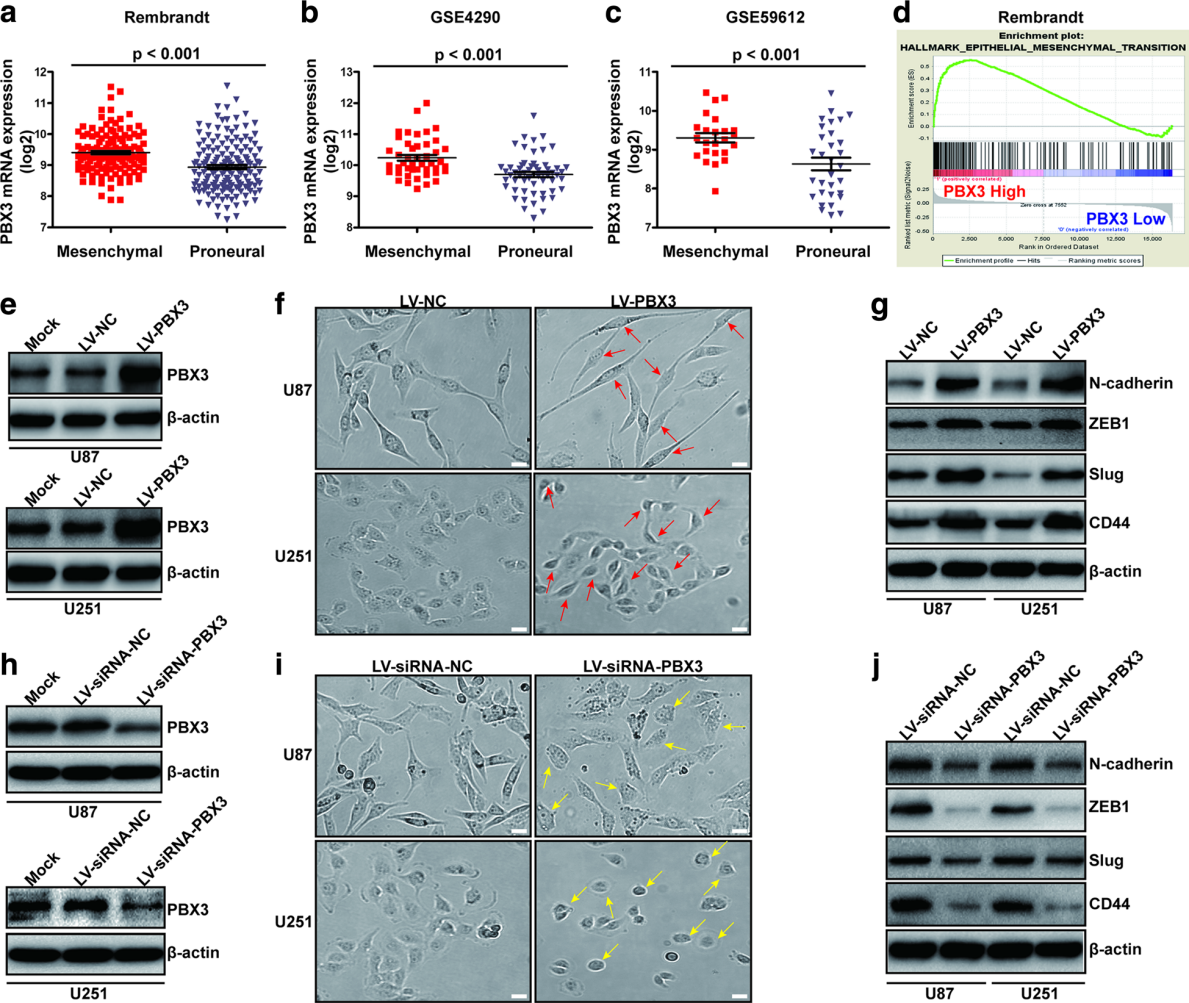
PBX3 is upregulated in mesenchymal gliomas and promotes glioma mesenchymal transition. **a-c** PBX3 expression is significantly higher in mesenchymal gliomas than that in proneural gliomas in Rembrandt (**a**), GSE4290 (**b**) and GSE59612 (**c**) datasets. **d** GSEA plot showed that hallmark of epithelial mesenchymal transition was significantly enriched in high PBX3 expression samples of GBM. **e** Verification of PBX3 overexpression by western blot assay. **f** The morphology of U87 and U251 cells infected with LV-NC or LV-PBX3 were compared under bright-field. Red arrows showed the morphological changes of U87 and U251 cells upon PBX3 overexpression. Scale bar = 100 μm. **g** Western blot analysis of N-cadherin, ZEB1, Slug and CD44 expressions upon PBX3 overexpression. **h** Verification of PBX3 knockdown by western blot assay. **i** The morphology of U87 and U251 cells infected with LV-siRNA-NC or LV-siRNA-PBX3 were compared under bright-field. Yellow arrows showed the morphological changes of U87 and U251 cells upon PBX3 knockdown. Scale bar = 100 μm. **j** Western blot analysis of N-cadherin, ZEB1, Slug and CD44 upon PBX3 knockdown

To evaluate the expression profiles of PBX3 between mesenchymal and proneural gliomas, mRNA data from Rembrandt, GSE4290 and GSE59612 were analyzed. Results showed that PBX3 expression was increased in mesenchymal gliomas compared with proneural gliomas (Fig. 1a-c). Using the expression data from the Rembrandt dataset, we performed GSEA to verify whether we could detect some hallmarks of cancer, including mesenchymal transition,in the patients with high-level of PBX3. Our results showed that hallmarks, including “Epithelial_Mesenchymal_Transition”, were significantly enriched in the patients with high-level of PBX3 (Fig. 1d). We chose four mesenchymal transition markers (N-cadherin, ZEB1, Slug, and CD44) from the gene list identified by GSEA, which are reported to be involved in glioma mesenchymal transition [6, 32–35], and analyzed the relationship between these markers and PBX3 expression. Pearson correlation analyses using mRNA data from Rembrandt dataset were performed. Results showed significant and positive correlations between PBX3 and N-cadherin, ZEB1, Slug and CD44 (Additional file 1: Figure S1A-D). To further confirm our observations, we measured PBX3 and mesenchymal markers mRNA levels in 45 GBM samples by qRT-PCR and observed high expression levels of N-cadherin, ZEB1, Slug and CD44 in GBMs with high PBX3 expression (*n* = 23) than those with low PBX3 expression (Additional file 1: Figure S1E-H). Having demonstrated that PBX3 is upregulated in mesenchymal gliomas and correlated with mesenchymal markers, we wondered whether PBX3 is required for maintenance of mesenchymal phenotype of GBM cells. To achieve this goal, we ectopically expressed PBX3 using lentiviral vectors and knocked down PBX3 expression using lentiviral siRNAs in U87 and U251 cells (Fig. 1e and h). Results showed that overexpression of PBX3 significantly promoted a fibroblast-like morphology and enhanced the expression of mesenchymal markers (Fig. 1 f and g). By contrast, knockdown of PBX3 triggered a cobblestone-like morphology and decreased the expression of mesenchymal markers (Fig. 1 i and j). On the basis of the mesenchymal promoting role of PBX3 in glioma, we focused on migration and invasion assays. As expected, PBX3 overexpression promoted glioma migration and invasion, while PBX3 downregulation inhibited glioma migration and invasion (Additional file 2: Figure S2). Collectively, these results indicated that PBX3 is associated with mesenchymal transition in gliomas.

### PBX3 is required for the TGF-β-induced mesenchymal transition

**Fig. 2:**
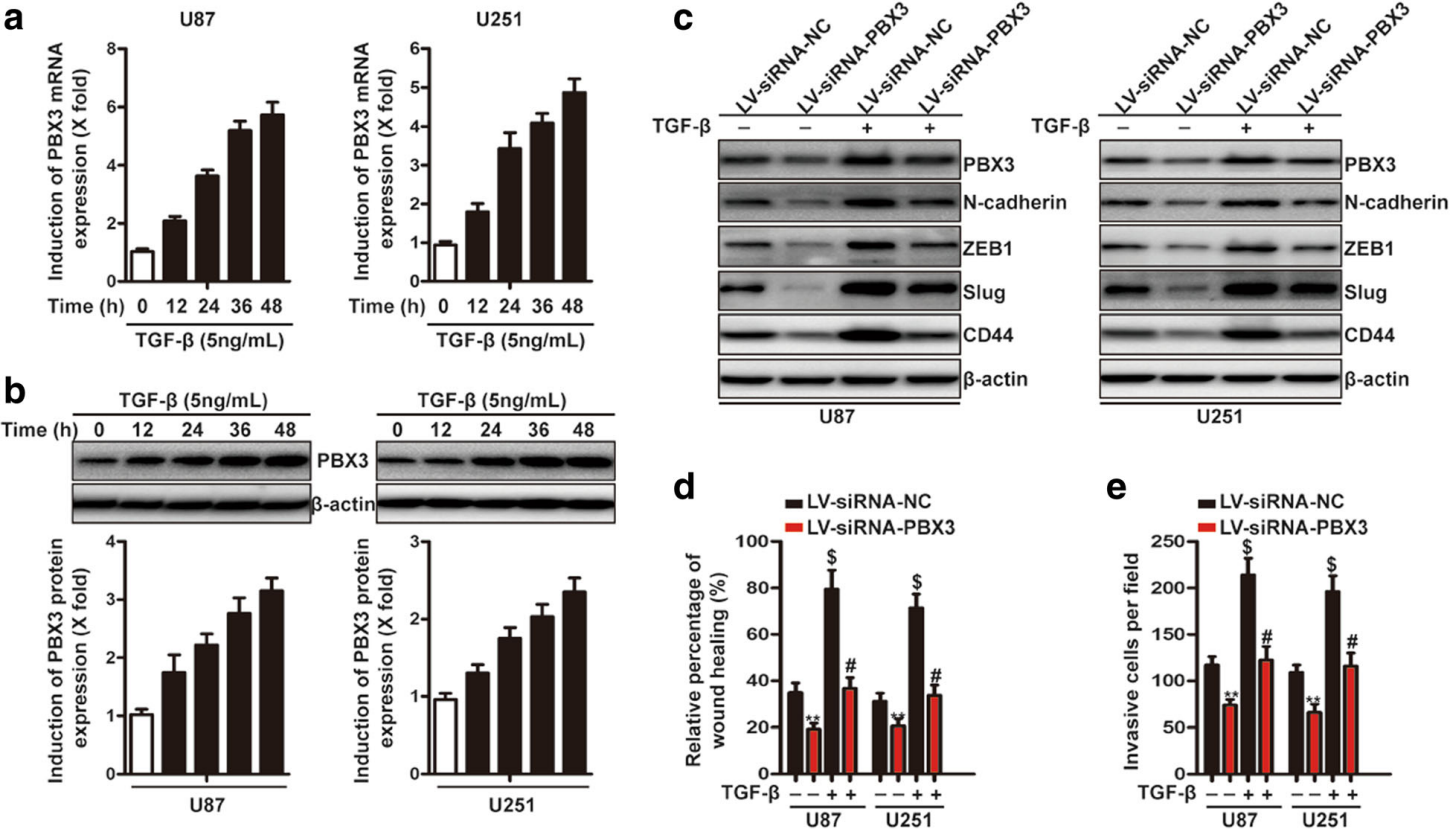
PBX3 is required for the induction of mesenchymal transition and cell migration and invasion in response to TGF-β. **a** qRT-PCR analysis of PBX3 expression in response to TGF-β (5 ng/mL) induction in U87 and U251 cells at different time points, as indicated. **b** Western blot analysis of PBX3 expression in response to TGF-β (5 ng/mL) induction in U87 and U251 cells at different time points, as indicated. **c** U87 and U251 cells stably expressing LV-siRNA-PBX3 or LV-siRNA-NC were treated with TGF-β for 48 h and immunoblotting analysis of PBX3, N-cadherin, ZEB1, Slug and CD44 were performed. **d** and **e** Quantification of wound-healing (**d**) and transwell assays (**e**). **indicates a statistical significant difference (*p* < 0.01) between LV-siRNA-NC + TGF-β (−) group and LV-siRNA-PBX3 + TGF-β (−) group. $ indicates a statistical significant difference (*p* < 0.01) between LV-siRNA-NC + TGF-β (−) group and LV-siRNA-NC + TGF-β (+) group. # indicates a statistical significant difference (*p* < 0.01) between LV-siRNA-NC + TGF-β (+) group and LV-siRNA-PBX3 + TGF-β (+) group

TGF-β is a master regulator of mesenchymal transition in GBM and induces the expression of mesenchymal genes, such as N-cadherin, ZEB1, Slug and CD44 [17, 36, 37]. Moreover, GSEA results showed that GBMs with high-level of PBX3 were enriched for the TGF-β signaling (Additional file 3: Figure S3). Therefore, we decided to explore the role of PBX3 in response to TGF-β. First, we examined the expression of PBX3 mRNA and protein upon exposure to TGF-β. The levels of PBX3 mRNA and protein in U87 and U251 cells were significantly upregulated during the 48-h post-incubation period (Fig. 2a and b). Next, we tested the effect of PBX3 downregulation on the TGF-β induced upregulation of N-cadherin, ZEB1, Slug and CD44. TGF-β induced upregulation of N-cadherin, ZEB1and CD44 were completely antagonized, whereas the accumulation of Slug was partially reversed by the knockdown of PBX3 (Fig. 2c). Finally, would-healing and transwell assays demonstrated that TGF-β-induced GBM cells migration and invasion were significantly decreased by PBX3 knockdown (Fig. 2d and e). In summary, these results demonstrated that PBX3 is required for the TGF-β-induced mesenchymal transition in GBM cells.

### LIN28/let-7b axis is required for PBX3-induced mesenchymal transition and for increased migration and invasion

**Fig. 3:**
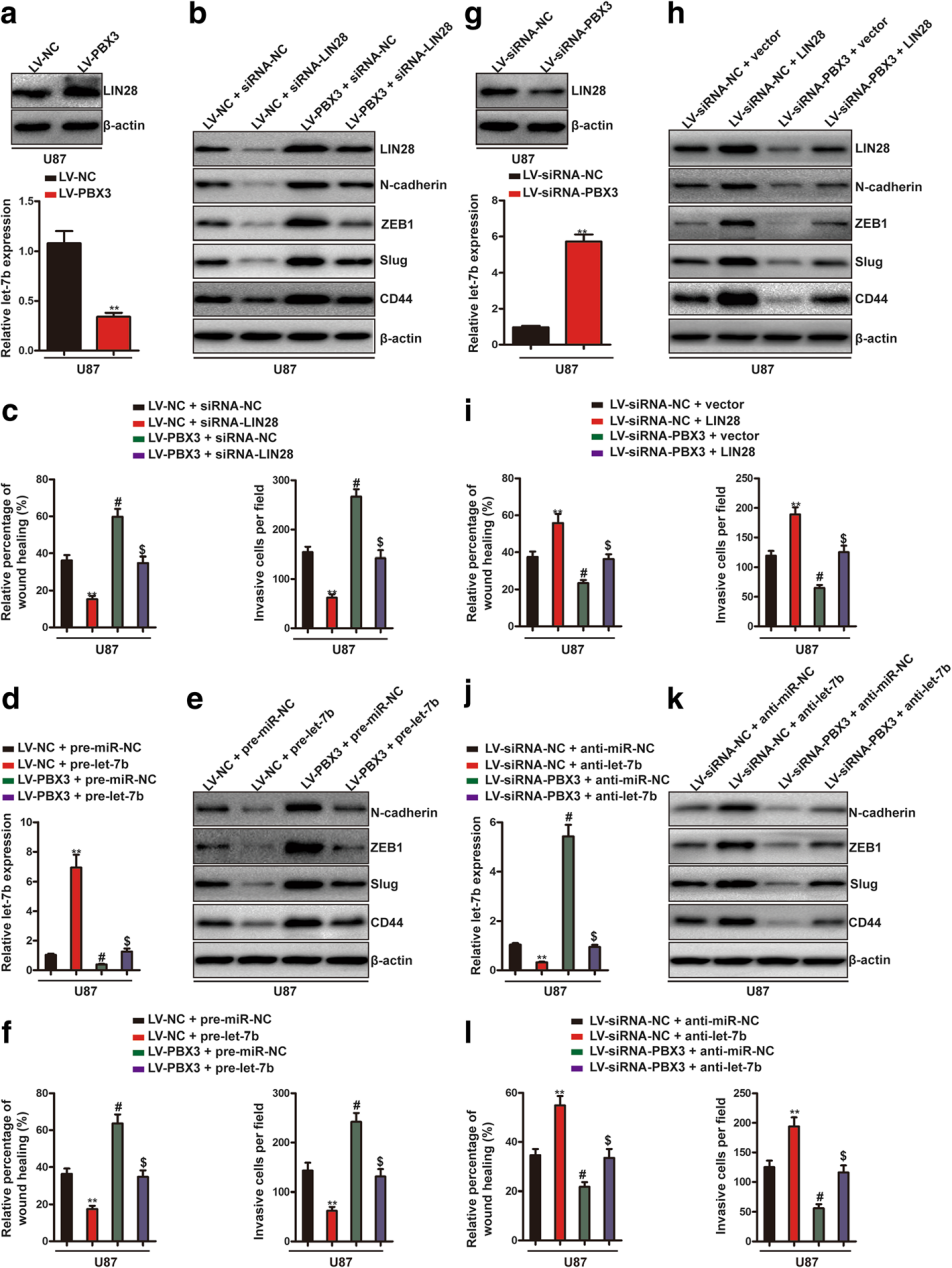
PBX3 promotes GBM mesenchymal transition, migration and invasion is mediated by LIN28/let-7b axis. **a** PBX3 overexpression remarkably upregulated LIN28 protein levels and downregulated let-7b expression in U87 cells. **b** U87 cells stably expressing LV-PBX3 or LV-NC were transfected with siRNA-NC or siRNA-LIN28 and then immunoblotting analysis of LIN28, N-cadherin, ZEB1, Slug and CD44 were performed. **c** Quantification of wound-healing (left) and transwell (right) assays. **indicates a statistical significant difference (*p* < 0.01) between LV-NC + siRNA-NC group and LV-NC + siRNA-LIN28 group. # indicates a statistical significant difference (*p* < 0.01) between LV-NC + siRNA-NC group and LV-PBX3 + siRNA-NC group. $ indicates a statistical significant difference (*p* < 0.01) between LV-PBX3 + siRNA-NC group and LV-PBX3 + siRNA-LIN28 group. **d** U87 cells stably expressing LV-PBX3 or LV-NC were transfected with pre-miR-NC or pre-let-7b and then let-7b expression were determined by qRT-PCR. **indicates a statistical significant difference (*p* < 0.01) between LV-NC + pre-miR-NC group and LV-NC + pre-let-7b group. # indicates a statistical significant difference (*p* < 0.01) between LV-NC + pre-miR-NC group and LV-PBX3 + pre-miR-NC group. $ indicates a statistical significant difference (*p* < 0.01) between LV-PBX3 + pre-miR-NC group and LV-PBX3 + pre-let-7b group. **e** U87 cells stably expressing LV-PBX3 or LV-NC were transfected with pre-miR-NC or pre-let-7b and then immunoblotting analysis of N-cadherin, ZEB1, Slug and CD44 were performed. **f** Quantification of wound-healing (left) and transwell (right) assays. **indicates a statistical significant difference (*p* < 0.01) between LV-NC + pre-miR-NC group and LV-NC + pre-let-7b group. # indicates a statistical significant difference (*p* < 0.01) between LV-NC + pre-miR-NC group and LV-PBX3 + pre-miR-NC group. $ indicates a statistical significant difference (*p* < 0.01) between LV-PBX3 + pre-miR-NC group and LV-PBX3 + pre-let-7b group. **g** PBX3 knockdown remarkably downregulated LIN28 protein levels and upregulated let-7b expression in U87 cells. **h** U87 cells stably expressing LV-siRNA-PBX3 or LV-siRNA-NC were transfected with LIN28 overexpressing plasmids or empty vectors and then immunoblotting analysis of LIN28, N-cadherin, ZEB1, Slug and CD44 were performed. **i** Quantification of wound-healing (left) and transwell (right) assays. **indicates a statistical significant difference (*p* < 0.01) between LV-siRNA-NC + vector group and LV-siRNA-NC + LIN28 group. # indicates a statistical significant difference (*p* < 0.01) between LV-siRNA-NC + vector group and LV-siRNA-PBX3 + vector group. $ indicates a statistical significant difference (*p* < 0.01) between LV-siRNA-PBX3 + vector group and LV-siRNA-PBX3 + LIN28 group. **j** U87 cells stably expressing LV-siRNA-PBX3 or LV-siRNA-NC were transfected with anti-miR-NC or anti-let-7b and then let-7b expression were determined by qRT-PCR. **indicates a statistical significant difference (*p* < 0.01) between LV-siRNA-NC + anti-miR-NC group and LV-siRNA-NC + anti-let-7b group. # indicates a statistical significant difference (*p* < 0.01) between LV-siRNA-NC + anti-miR-NC group and LV-siRNA-PBX3 + anti-miR-NC group. $ indicates a statistical significant difference (*p* < 0.01) between LV-siRNA-PBX3 + anti-miR-NC group and LV-siRNA-PBX3 + anti-let-7b group. **k** U87 cells stably expressing LV-siRNA-PBX3 or LV-siRNA-NC were transfected with anti-miR-NC or anti-let-7b and then immunoblotting analysis of N-cadherin, ZEB1, Slug and CD44 were performed. **l** Quantification of wound-healing (left) and transwell (right) assays. **indicates a statistical significant difference (*p* < 0.01) between LV-siRNA-NC + anti-miR-NC group and LV-siRNA-NC + anti-let-7b group. # indicates a statistical significant difference (*p* < 0.01) between LV-siRNA-NC + anti-miR-NC group and LV-siRNA-PBX3 + anti-miR-NC group. $ indicates a statistical significant difference (*p* < 0.01) between LV-siRNA-PBX3 + anti-miR-NC group and LV-siRNA-PBX3 + anti-let-7b group

To confirm our hypothesis that PBX3 promotes glioma mesenchymal transition and then triggers migration and invasion via activating LIN28/let-7 axis, we first measured the expression of LIN28 and let-7 upon PBX3 overexpression. Results showed that overexpression of PBX3 increased LIN28 protein levels while decreased let-7 levels, with let-7b being the most downregulated one (Fig. 3a, Additional file 4: Figure S4 and Additional file 5: Figure S5A), suggesting that PBX3 positively regulates LIN28/let-7 axis. As let-7b represents the most significantly downregulated let-7 family microRNAs upon PBX3 overexpression, we selected let-7b for the following experiments. Next, we tested whether PBX3-driven GBM mesenchymal phenotype was dependent on LIN28/let-7b axis. LIN28 knockdown prevented PBX3-mediated increases in cell migration and invasion and reserved PBX3-induced mesenchymal phenotype (Fig. 3b and c and Additional file 5: Figure S5B and C). Consistently, overexpression of let-7b reversed PBX3-driven GBM cell migration, invasion and mesenchymal transition (Fig. 3d-f and Additional file 5: Figure S5D-F). To further confirm our hypothesis, we also measured LIN28 and let-7b expression under PBX3 knockdown conditions. Results showed that downregulation of PBX3 decreased LIN28 protein levels and increased let-7b expressions(Fig. 3g and Additional file 5: Figure S5G). As expected, overexpression of LIN28 or inhibition of let-7b expression reversed PBX3 downregulation-induced decreased cell migration, invasion and mesenchymal phenotype (Fig. 3h-l and Additional file 5: Figure S5H-L). Collectively, these results demonstrated that LIN28/let-7b axis is required for PBX3-driven mesenchymal transition and for increased migration and invasion.

### PBX3 activates LIN28/let-7b axis via ERK1/2 and c-myc-dependent mechanisms

**Fig. 4:**
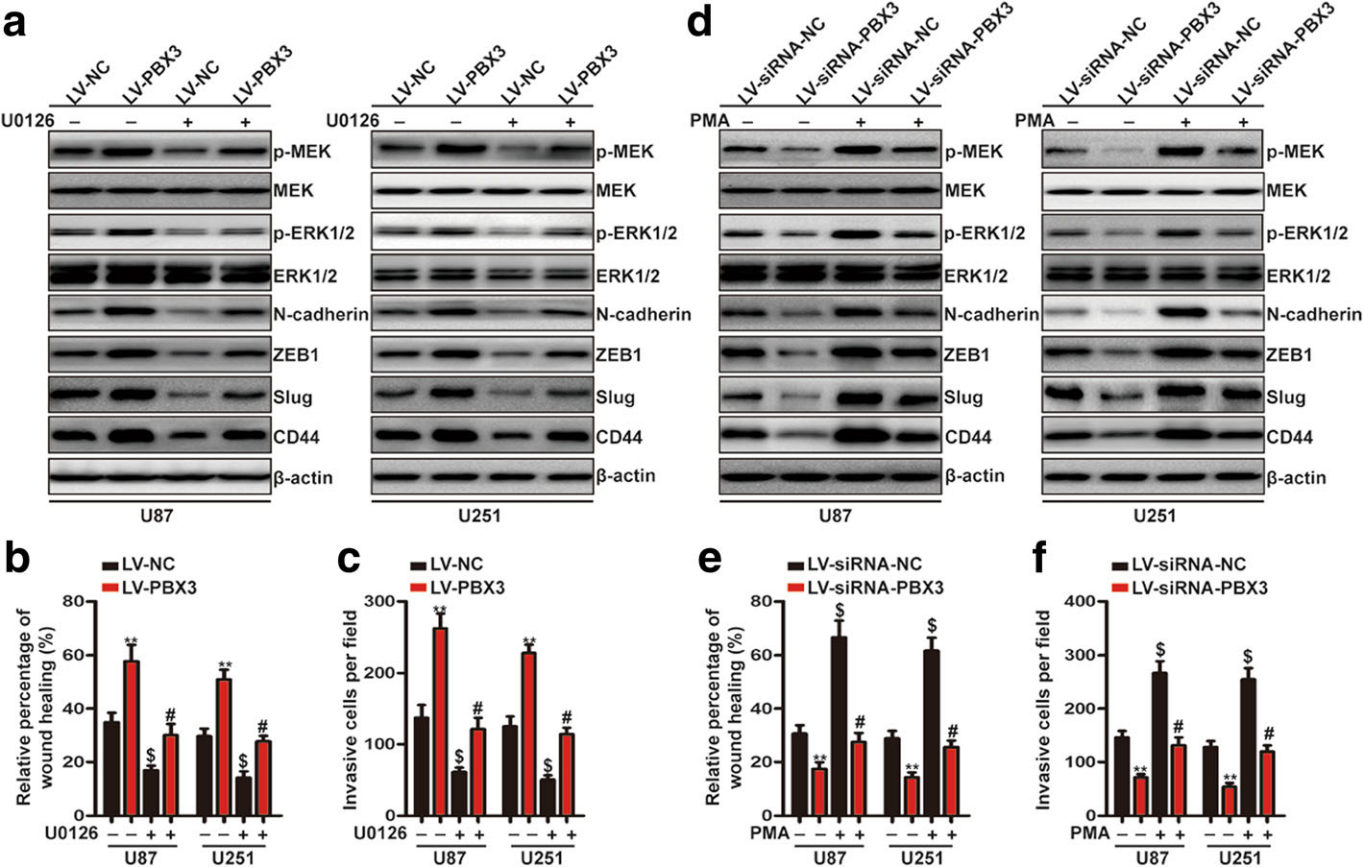
PBX3 promotes GBM mesenchymal transition, migration and invasion via activating MEK/ERK1/2 signaling pathway. **a** U87 and U251 cells stably expressing LV-PBX3 or LV-NC were treated with U0126 for 24 h and immunoblotting analysis of p-MEK, MEK, p-ERK1/2, ERK1/2, N-cadherin, ZEB1, Slug and CD44 were performed. **b** and **c** Quantification of wound-healing (**b**) and transwell (**c**) assays. **indicates a statistical significant difference (*p* < 0.01) between LV-NC + U0126 (−) group and LV-PBX3 + U0126 (−) group. $ indicates a statistical significant difference (*p* < 0.01) between LV-NC + U0126 (−) group and LV-NC + U0126 (+) group. # indicates a statistical significant difference (*p* < 0.01) between LV-NC + U0126 (+) group and LV-PBX3 + U0126 (+) group. **d** U87 and U251 cells stably expressing LV-siRNA-PBX3 or LV-siRNA-NC were treated with PMA for 24 h and immunoblotting analysis of p-MEK, MEK, p-ERK1/2, ERK1/2, N-cadherin, ZEB1, Slug and CD44 were performed. **e** and **f** Quantification of wound-healing (**e**) and transwell (**f**) assays. **indicates a statistical significant difference (*p* < 0.01) between LV-siRNA-NC + PMA (−) group and LV-siRNA-PBX3 + PMA (−) group. $ indicates a statistical significant difference (*p* < 0.01) between LV-siRNA-NC + PMA (−) group and LV-siRNA-NC + PMA (+) group. # indicates a statistical significant difference (*p* < 0.01) between LV-siRNA-NC + PMA (+) group and LV-siRNA-PBX3 + PMA (+) group

Having demonstrated that PBX3 activates LIN28/let-7b pathway in GBM, we next investigated whether PBX3 regulates LIN28/let-7b axis via ERK1/2-dependent mechanism as our previously expected. To achieve this goal, we need to investigate whether MEK/ERK1/2 pathway mediated PBX3-driven GBM mesenchymal transition. First, we determined the changes in expression of phospho-MEK, MEK, phospho-ERK1/2 and ERK1/2 in GBM cells with altered expression of PBX3. As expected, phospho-MEK and phospho-ERK1/2 expression were increased after overexpression of PBX3 and decreased upon PBX3 downregulation (Fig. 4a and d). These data clearly demonstrated that PBX3 could activate MEK/ERK1/2 pathway in GBM cells. Next, we inhibited MEK/ERK1/2 activation in PBX3-overexpressed cells by U0126 and activated MEK/ERK1/2 pathway in PBX3-downregulated cells by PMA to determine whether PBX3-mediated GBM cell migration, invasion and mesenchymal transition were dependent on MEK/ERK1/2 pathway. As shown in Fig. 4a, the upregulation of phospho-MEK and phospho-ERK1/2 expression by PBX3 overexpression were significantly reduced by U0126 incubation. In the meantime, the promotive effects of PBX3 on cell migration, invasion and mesenchymal transition were markedly reversed by U0126 treatment (Fig. 4a-c). Consistently, the decreased expression of phospho-MEK and phospho-ERK1/2 expression by PBX3 downregulation were restored by PMA treatment. Meanwhile, the inhibitory effects after PBX3 downregulation on cell migration, invasion and mesenchymal transition were reversed by PMA incubation (Fig. 4d-f). These results confirmed the hypothesis that PBX3 promotes GBM migration, invasion and mesenchymal transition via activation of MEK/ERK1/2 pathway.

**Fig. 5:**
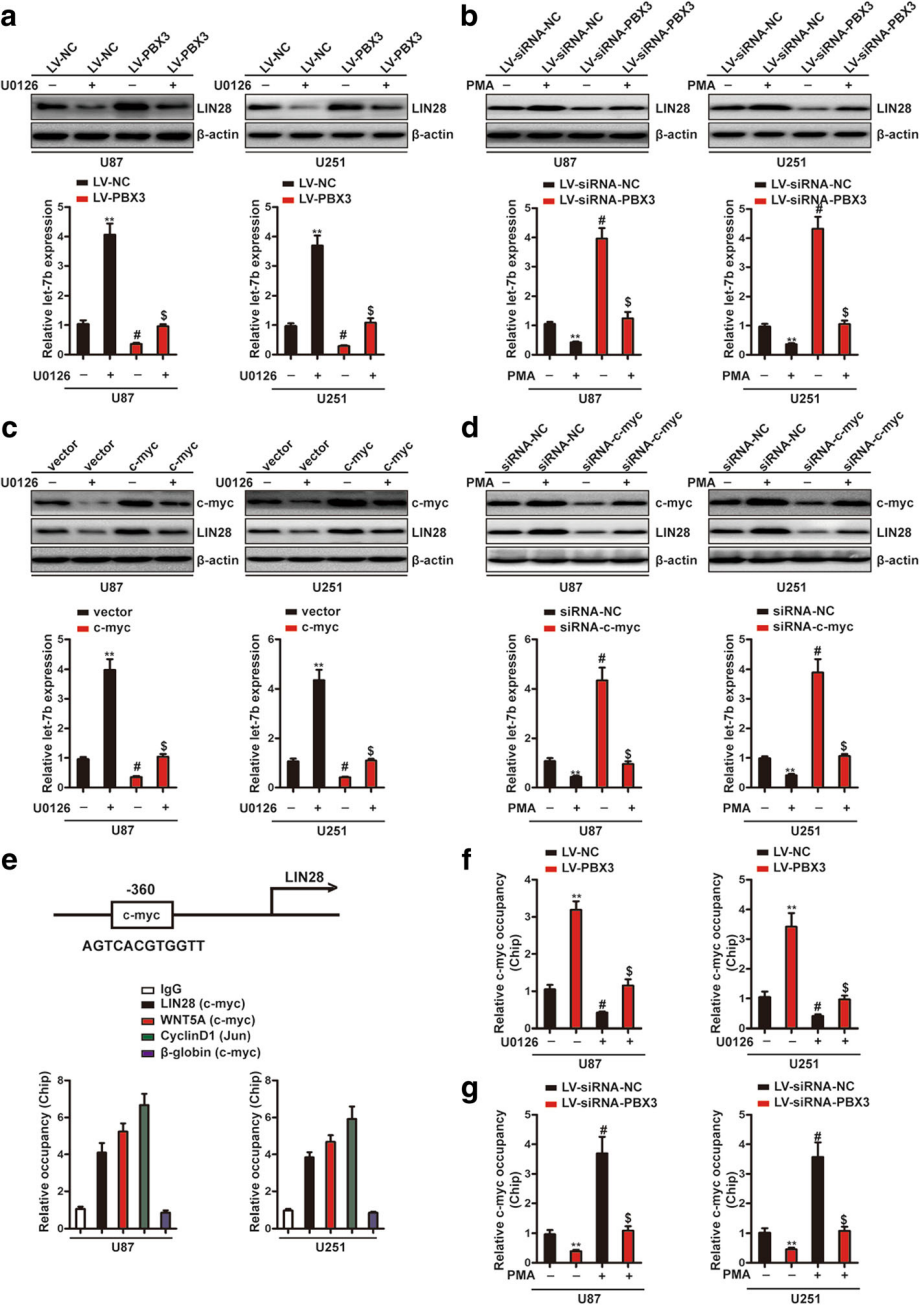
PBX3 activates LIN28/let-7b axis via ERK1/2 and c-myc-dependent mechanisms. **a** GBM cells stably expressing LV-PBX3 or LV-NC were treated with U0126 for 24 h and then LIN28 protein levels and let-7b expression were determined by western blot and qRT-PCR assays, respectively. **indicates a statistical significant difference (*p* < 0.01) between LV-NC + U0126 (−) group and LV-NC + U0126 (+) group. # indicates a statistical significant difference (*p* < 0.01) between LV-NC + U0126 (−) group and LV-PBX3 + U0126 (−). $ indicates a statistical significant difference (*p* < 0.01) between LV-PBX3 + U0126 (−) group and LV-PBX3 + U0126 (+) group. **b** GBM cells stably expressing LV-siRNA-PBX3 or LV-siRNA-NC were treated with PMA for 24 h and then LIN28 protein levels and let-7b expression were determined by western blot and qRT-PCR assays, respectively. **indicates a statistical significant difference (*p* < 0.01) between LV-siRNA-NC + PMA (−) group and LV-siRNA-NC + PMA (+) group. # indicates a statistical significant difference (*p* < 0.01) between LV-siRNA-NC + PMA (−) group and LV-siRNA-PBX3 + PMA (−). $ indicates a statistical significant difference (*p* < 0.01) between LV-siRNA-PBX3 + PMA (−) group and LV-siRNA-PBX3 + PMA (+) group. **c** GBM cells infected with c-myc overexpressing plasmids or empty vectors were treated with U0126 for 24 h and then LIN28 and c-myc protein levels were determined by western blot assays. Let-7b expression were measured by qRT-PCR assays. **indicates a statistical significant difference (*p* < 0.01) between vector + U0126 (−) group and vector + U0126 (+) group. # indicates a statistical significant difference (*p* < 0.01) between vector + U0126 (−) group and c-myc + U0126 (−). $ indicates a statistical significant difference (*p* < 0.01) between c-myc + U0126 (−) group and c-myc + U0126 (+) group. **d** GBM cells infected with siRNA-NC or siRNA-c-myc were treated with PMA for 24 h and then LIN28 and c-myc protein levels were determined by western blot assays. Let-7b expression were measured by qRT-PCR assays. **indicates a statistical significant difference (*p* < 0.01) between siRNA-NC + PMA (−) group and siRNA-NC + PMA (+) group. # indicates a statistical significant difference (*p* < 0.01) between siRNA-NC + PMA (−) group and siRNA-c-myc + PMA (−). $ indicates a statistical significant difference (*p* < 0.01) between siRNA-c-myc + PMA (−) group and siRNA-c-myc + PMA (+) group. **e** c-myc regulates LIN28 expression by binding to its promoter in GBM cells. Diagram of amplicons for ChIP-qPCR were illustrated. ChIPs were performed with anti-c-myc antibody and anti-Jun antibody (a positive control). ChIP was analyzed by qRT-PCR, with primers in the LIN28, WNT5A (a positive control), CyclinD1 (a positive control), and β-globin (a negative control) promoters. **f** U0126 treatment reversed the effect of PBX3 overexpression-induced enhanced c-myc binding to LIN28 promoters. **indicates a statistical significant difference (*p* < 0.01) between LV-NC + U0126 (−) group and LV-PBX3 + U0126 (+) group. # indicates a statistical significant difference (*p* < 0.01) between LV-NC + U0126 (−) group and LV-NC + U0126 (+). $ indicates a statistical significant difference (*p* < 0.01) between LV-PBX3 + U0126 (−) group and LV-PBX3 + U0126 (+) group. **g** PMA treatment reversed the effect of PBX3 knockdown-induced decreased c-myc binding to LIN28 promoters. **indicates a statistical significant difference (*p* < 0.01) between LV-siRNA-NC + PMA (−) group and LV-siRNA-PBX3 + PMA (+) group. # indicates a statistical significant difference (*p* < 0.01) between LV-siRNA-NC + PMA (−) group and LV-siRNA-NC + PMA (+). $ indicates a statistical significant difference (*p* < 0.01) between LV-siRNA-PBX3 + PMA (−) group and LV-siRNA-PBX3 + PMA (+) group

To demonstrate that PBX3 regulates LIN28/let-7b axis via ERK1/2-dependent mechanism. We inhibited ERK1/2 signaling in PBX3-overexpressing cells and activated ERK1/2 signaling in PBX3-knockdown cells. As shown in Fig. 5a and b, ERK1/2 inhibition reversed PBX3 overexpression induced LIN28 upregulation and let-7b downregulation, while ERK1/2 activation restored PBX3 depletion induced LIN28 downregulation and let-7b upregulation. Thus, these data suggest that PBX3 promotes LIN28/let-7b axis via ERK1/2-dependent mechanism. Previous studies have demonstrated that ERK1/2 regulates LIN28/let-7b axis via c-myc-dependent way and c-myc regulates LIN28 expression by directly binding to its promoter [30, 38]. As c-myc plays central roles in GBM progression, including mesenchymal transition [39], we proposed that ERK1/2 signaling promotes LIN28/let-7b axis in GBM cells via c-myc-dependent pathway. To test whether the hypothesis is true and whether the transcriptional factor c-myc might mediated PBX3/ERK1/2-driven mesenchymal transition, we overexpressed c-myc in ERK1/2-inhibited cells and knocked down c-myc in ERK1/2-activated cells. Results showed that LIN28 downregulation and let-7b upregulation induced by ERK1/2 inhibition were prevented by c-myc overexpression (Fig. 5c). Consistently, LIN28 upregulation and let-7b downregulation induced by ERK1/2 activation were reversed by c-myc knockdown (Fig. 5d). Furthermore, ChIP assays confirmed previous studies that c-myc can directly bind to the promoter of LIN28 and this biding was depended on ERK1/2 signaling (Fig. 5e-g). Finally, we found that silencing c-myc can reduced PBX3 overexpression induced increased migration, invasion and mesenchymal transition, while overexpression of c-myc can restore PBX3 inhibition induced decreased migration, invasion and mesenchymal transition (Additional file 6: Figure S6). Collectively, our finding demonstrated that PBX3 activates LIN28/let-7b axis in GBM cells via ERK1/2-c-myc-dependent mechanism.

### Let-7b directly targets PBX3 3′-UTR and forms a positive feedback loop

**Fig. 6:**
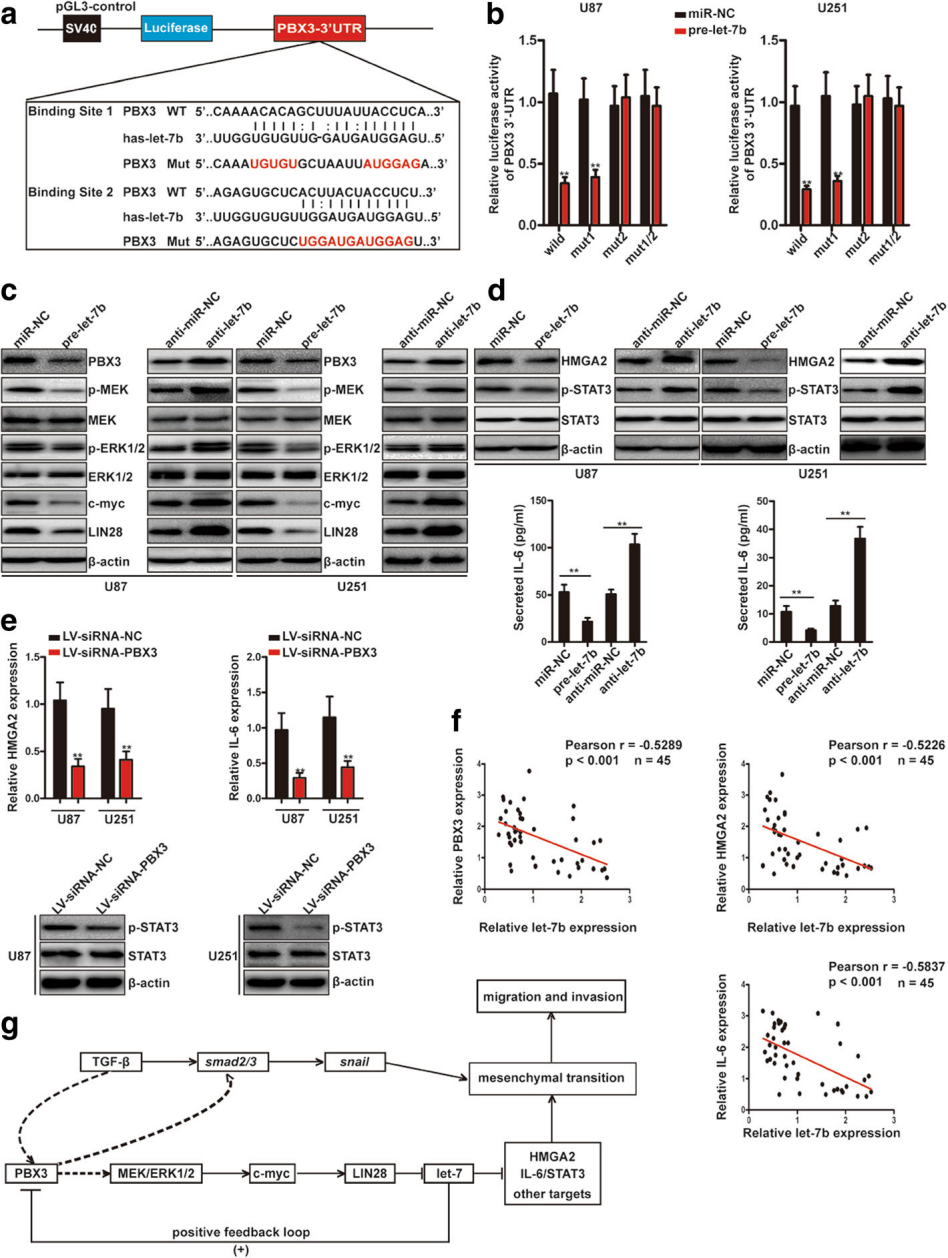
Let-7b directly targets PBX3 3′-UTR and forms a positive feedback loop. **a** Predicted binding sites of wild-type (WT) and mutant sequences of let-7b in the 3′-UTR of PBX3 mRNA. **b** Luciferase reporter assays were performed in U87 and U251 cells with co-transfection of indicated WT or mutant 3′-UTR constructs and pre-let-7b mimic or miR-NC mimic. **p < 0.01. **c** Western blot analysis of PBX3, p-MEK, MEK, p-ERK1/2, ERK1/2, c-myc, and LIN28 protein levels in let-7b overexpressing- or depleting-cells. **d** Western blot analysis of HMGA2, p-STAT3, and STAT3 protein levels in let-7b overexpressing- or depleting-cells and ELISA analysis of IL-6 protein levels in the supernatants of let-7b overexpressing- or depleting-cells. **p < 0.01. **e** qRT-PCR analysis of HMGA2 and IL-6 mRNA levels and western blot analysis of p-STAT3 and STAT3 protein levels in U87 and U251 cells transfected with LV-siRNA-NC or LV-siRNA-PBX3. **f** Pearson’s correlation analyses indicated that let-7b expression was negatively associated with PBX3, HMGA2 and IL-6 mRNA levels in GBM tissues. **g** Schematic diagram of the positive feedback loop involves PBX3, MEK/ERK1/2, c-myc and LIN28/let-7b

Previous studies have demonstrated that PBX3 can be negatively regulated by various miRNAs, including let-7 family members (miR-98, let-7c, and let-7d) [19, 20, 26]. However, whether PBX3 can be regulated by let-7b in GBM remains undetermined. To test whether PBX3 is a direct target of let-7b, luciferase report assays were performed. Fig. 6a shows the predicted interaction between let-7b and the target sites. Relative luciferase activity was significantly inhibited by let-7b when the PBX3 plasmid containing the wild 3′-UTR and mut1 3′-UTR were present. However, mutations in binding site 2 (mut2) or both the binding sites were mutated abrogated the suppressive effect of let-7b (Fig. 6b). Moreover, western blot analysis showed that let-7d suppressed PBX3 and the downstream targets MEK/ERK1/2, c-myc and LIN28. In contrast, suppression of let-7b enhanced PBX3 and its downstream signaling (Fig. 6c). Taken together, these results suggest that PBX3 is a bona fide target of let-7b and there exists a positive feedback loop between PBX3 and let-7b. Moreover, other targets of let-7 family, which are mesenchymal transition relevant factors, such as HMGA2 and IL-6/STAT3, were also evaluated in our present study. As shown in Fig. 6d, let-7b can also inhibit HMGA2 and IL-6/STAT3 pathways. As PBX3 regulates LIN28/let-7b axis, we wondered whether PBX3 regulates let-7b targets in glioma cells. Therefore, we tested the effect of PBX3 silencing on the expression of let-7b targets. As shown in Fig. 6e, PBX3 silencing decreased the mRNA levels of HMGA2 and IL-6 and protein ratios of p-STAT3/STAT3 in both U87 and U251 cells. Indeed, our GSEA results showed that hallmark of “IL-6_JAK_STAT3 signaling” is significantly enriched in the patients with high-level of PBX3 (Additional file 7: Figure S7).

We further measured let-7b, HMGA2 and IL-6 mRNA levels in 45 GBM samples and Pearson’s correlation analyses were performed. Results showed that let-7b inversely correlated with PBX3, HMGA2 and IL-6 expression (Fig. 6f), supporting that PBX3, HMGA2 and IL-6 are targets of let-7b. Until now, we established a positive feedback loop between PBX3 and let-7b, which involves MEK/ERK1/2, c-myc and LIN28, and ultimately affects HMGA2 and IL-6/STAT3 pathways to promotes GBM mesenchymal transition, migration and invasion (Fig. 6g).

### Downregulation of PBX3 inhibits GBM invasion in vivo

**Fig. 7:**
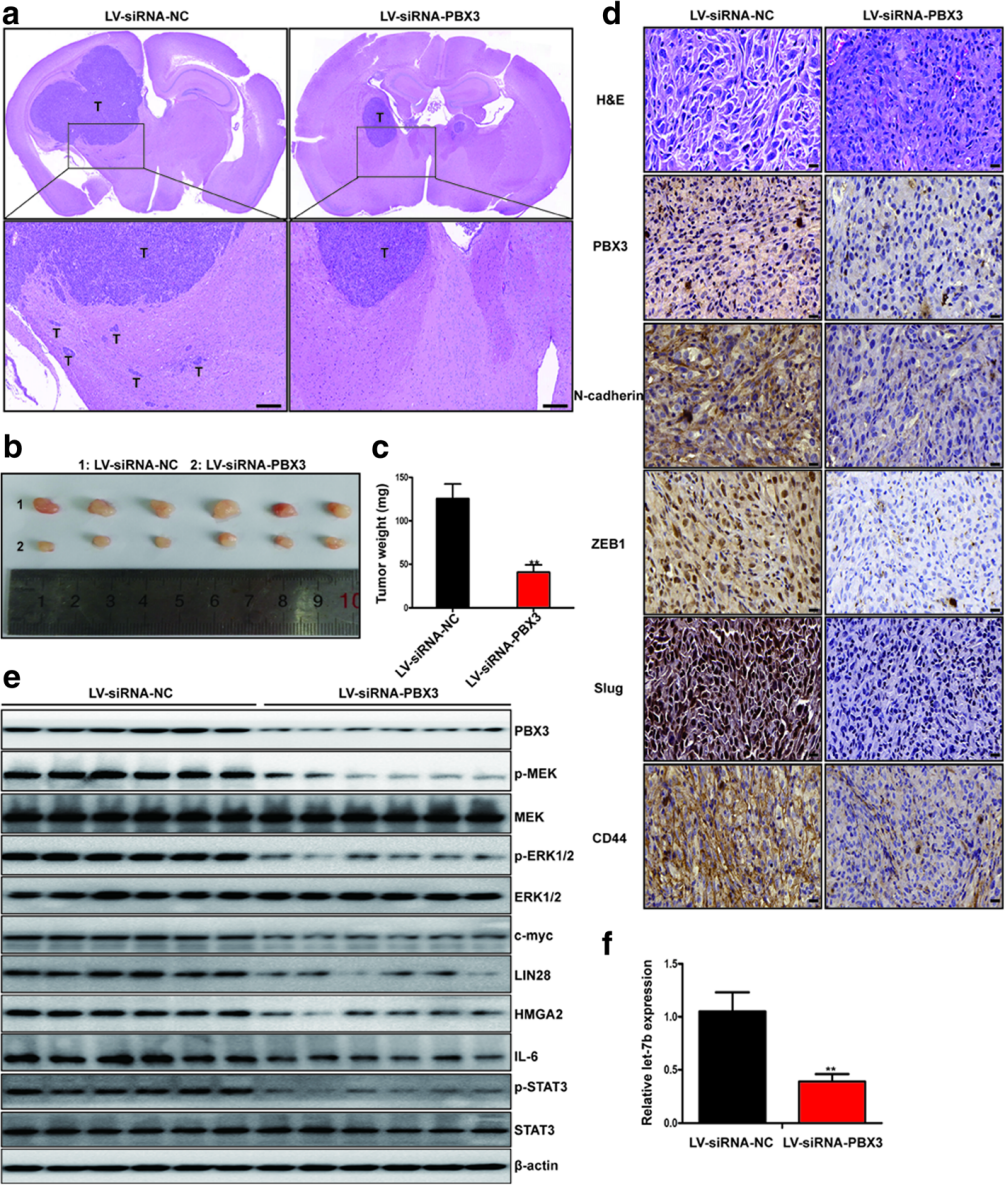
Targeting PBX3 inhibits GBM invasion in vivo. **a** Representative images of Hematoxylin and eosin (H&E) staining of tissues from mice with orthotopic tumors derived from LV-siRNA-NC-U87 or LV-siRNA-PBX3-U87 cells. Scale bar = 500 μm. **b** Images of orthotopic tumors derived from LV-siRNA-NC-U87 or LV-siRNA-PBX3-U87 cells. **c** The tumors derived from LV-siRNA-NC-U87 or LV-siRNA-PBX3-U87 cells were weighed after imaging. **p < 0.01. **d** H&E staining and immunohistochemistry for PBX3, N-cadherin, ZEB1, Slug and CD44 in tumors derived from LV-siRNA-NC-U87 or LV-siRNA-PBX3-U87 cells. Scale bar = 50 μm. **e** Western blot analysis of PBX3, p-MEK, MEK, p-ERK1/2, ERK1/2, c-myc, LIN28, IL-6, p-STAT3, and STAT3 in tumors derived from LV-siRNA-NC-U87 or LV-siRNA-PBX3-U87 cells. **f** qRT-PCR analysis of let-7b expression in tumors derived from LV-siRNA-NC-U87 or LV-siRNA-PBX3-U87 cells. **p < 0.01

To further confirm the role of PBX3 in GBM invasion and assess the therapeutic potential of PBX3 depletion in vivo, LV-siRNA-NC- and LV-siRNA-PBX3-transfected U87 cells were intracranially injected into nude mice. As shown in Fig. 7a, downregulation of PBX3 consistently led to decreased tumor invasion compared with the control group. In addition, PBX3 depletion reduced tumor growth and resulted in the formation of significantly smaller tumors when compared with the control group (Fig. 7b and c). These results demonstrated that targeting PBX3 inhibits GBM invasion and growth in vivo. Next, we performed PBX3, N-cadherin, ZEB1, Slug and CD44 staining to assess the effects of PBX3 on GBM mesenchymal transition. Results showed that inhibition of PBX3 suppressed the expression of N-cadherin, ZEB1, Slug and CD44 (Fig. 7d), indicating that downregulation of PBX3 inhibits GBM mesenchymal transition in vivo. Finally, we investigated whether MEK/ERK1/2/c-myc/LIN28/let-7b pathway was suppressed upon PBX3 knockdown. Our western blot and qRT-PCR results demonstrated that PBX3 knockdown inhibited p-MEK, p-ERK1/2, c-myc, LIN28, HMGA2, IL-6 and p-STAT3 protein levels and increased let-7b expression (Fig. 7e and f). Taken together, these data support our in vitro findings and reinforce the hypothesis that PBX3 has a critical role in the maintenance of a mesenchymal and invasive phenotype in human GBM.

## Discussion

Despite aggressive treatment, the prognosis of patients with GBM remains dismal, which is partly caused by the highly invasive nature of GBM [4]. Recently, mesenchymal transition of GBM has been recognized as a key driver of GBM invasion and malignant progression, but signals promote this process are still unclear [7]. Here, we presents evidence for a role of PBX3 in the regulation of the mesenchymal transition program that maintains invasive phenotypes of human GBM. Our demonstrations that PBX3 is upregulated in mesenchymal gliomas compared with proneural gliomas and that it positively correlated with several mesenchymal markers highlight the significance of PBX3 in mesenchymal transition. Notably, we showed that PBX3 actives MEK/ERK1/2 axis, which negatively regulates let-7b by inducing LIN28 expression through oncogenic c-myc transcription. In turn, let-7b inhibits PBX3 expression by directly targeting the 3′-UTR of PBX3. Thus, these data implicate PBX3, MEK/ERK1/2, c-myc and LIN28/let-7b in a positive feedback loop. In addition, PBX3 has been demonstrated to be required for TGF-β-induced mesenchymal transition, suggesting that this positive feedback loop may be a part of TGF-β signaling. Based on these findings, we proposed that PBX3 could be a promising therapeutic target for preventing GBM mesenchymal transition and invasion.

The members of the PBX family have vital roles in both development and differentiation through regulating gene transcription [40, 41]. Dysregulation of PBX family members has been implicated in various types of human cancers, including GBM [22, 26, 27]. Although our previous studies suggested a role for PBX3 in the biology of GBM proliferation, cell cycle progression, migration and invasion [26, 27], there is a paucity of data defining the functional role that PBX3 plays in GBM mesenchymal transition. Herein, we first showed that PBX3 is upregulated in mesenchymal gliomas and positively correlated with mesenchymal markers, such as N-cadherin, ZEB1, Slug and CD44. These results suggest that an aberrant increase in PBX3 expression is linked to GBM mesenchymal transition. Furthermore, functional studies showed that PBX3 expression directly impacts GBM invasive phenotypes and mesenchymal transition, as measured by in vitro and in vivo experiments. Our findings is consistent with previous reports that PBX3 functions as an oncogene and EMT enhancer in other cancers [21, 42, 43].

Although the mesenchymal transition promoting role of PBX3 in GBM has been established, detailed molecular mechanism mediated the role of PBX3 remains largely unknown. It was recently proposed that PBX3 is a regulator of MEK/ERK1/2 pathway [25]. As activation of MEK/ERK1/2 pathway usually facilitates GBM mesenchymal transition and progression [44], it is reasonable to hypothesis that PBX3 promotes GBM mesenchymal transition via activation of MER/ERK1/2. Indeed, we demonstrated that PBX3 promotes GBM mesenchymal transition was mediated by MEK/ERK1/2 pathway. However, how PBX3 activates MEK/ERK1/2 in GBM remains largely unknown. Several activators of MEK/ERK1/2 in human cancers have been identified, such as RAF1 [45], IGF-1R [46] and Connexin-43 [47]. Whether PBX3 activates MEK/ERK1/2 to promote mesenchymal transition through these driving factors remains to be investigated in future.

LIN28 has been shown to be a key RNA-binding protein and plays a critical role in cellular reprogramming and tumor transformation [48]. Overexpression of LIN28 was prevalent in human cancers, including GBM [28]. Moreover, through inhibiting let-7 biogenesis, LIN28 influences let-7 targets translation and promotes malignant progression of cancers [49]. Recent reports showed that the LIN28/let-7 axis plays a critical role in promoting the cancer development, Warburg effect and cancer stem-cell like cells [50–52]. However, the detailed molecular mechanisms involved in regulating LIN28/let-7 pathway in GBM is still unclear. Several mechanisms that contribute to the dysregulated LIN28 in cancers have been identified in previous studies. For example, miRNAs, such as miR-125b, was reported to inhibit LIN28 in some embryonic stem cells as well as in glioma cells [17, 53]. Of note, let-7 miRNAs themselves have also been reported to regulate LIN28 expression, in a feedback manner [50]. Moreover, several transcriptional factors, such as NF-κB [54], c-myc [30] and β-catenin [55], can activate LIN28 and repress let-7 expression to augment cancer progression, while REST and ESE3/EHF are transcriptional repressors of LIN28 [49, 51]. In our present study, we showed that PBX3 can indirectly regulate LIN28 by activating MEK/ERK1/2 axis and then inducing LIN28 expression by transcription factor c-myc, which is consistent with previous reports [30]. The crosstalk between PBX3 and other LIN28 regulators, such as NF-κB, β-catenin, REST and ESE3/EHF should be investigated in future.

Recent studies reported that c-myc may play a key regulatory role in promoting EMT in many types of cancers [56]. Importantly, c-myc has been reported to regulate miRNAs, which mediate its functions in cancer progression, including EMT. For example, Shao et al. reported that c-myc posttranscriptionally upregulates the expression of PHD finger protein 8 by repressing miR-22 to promote breast cancer EMT [57]. In this study, we demonstrated that c-myc mediates PBX3 induced GBM mesenchymal transition at least partially by repressing let-7b expression. Previous studies have also documented that c-myc inhibits let-7 expression by upregulating let-7 biogenesis inhibitor LIN28 [30], which is consistent with our result. Other miRNAs regulated by c-myc, such as miR-9, miR-23a, miR-15a/16–1, miR-106b, miR-34a, miR-148a and miR-25, are also involved in EMT regulation [58]. Thus, targeting c-myc-induced miRNAs or restoring the expression of c-myc repressed miRNAs seems to be an obvious strategy to combat c-myc-driven EMT. Additional studies will be necessary to identify miRNAs connected to c-myc in GBM progression, especially in GBM mesenchymal transition, for targeted therapy.

TGF-β is a master inducer of EMT in cancer cells [59]. In this study, our data indicate that PBX3 represent an important modulator of cell responsiveness to TGF-β: PBX3 is necessary for the induction of the mesenchymal transition in response to TGF-β in GBM cells. However, the molecular mechanisms by which PBX3 modulates the cellular sensitivity to TGF-β remains undetermined in this study. Previous studies have demonstrated that small mother against decapentaplegic (SMAD) signaling elicited by TGF-β plays a critical role in TGF-β induced EMT [60]. In addition, activated TGF-β signaling pathway and high levels of phosphorylation-SMADs were found in human gliomas [61]. Whether PBX3 mediates TGF-β-induced GBM mesenchymal transition through regulating SMAD signaling will be investigated in our next paper.

## Conclusions

In summary, we identified a PBX3/MEK/ERK1/2/c-myc/LIN28/let-7b positive feedback loop in GBM cells that induces GBM mesenchymal transition and promotes invasive phenotypes. Thus, PBX3 may serve as a target for prevention and treatment of GBM progression.

## Additional files

Additional file 1: **Figure S1.** PBX3 is positively correlated with mesenchymal markers. (A-D) Pearson’s correlation analyses indicate that PBX3 expression is positively associated with N-cadherin (A), ZEB1 (B), Slug (C), and CD44 (D) expressions in Rembrandt database. (E-H) The expression of N-cadherin (E), ZEB1 (F), Slug (G), and CD44 (H) are increased in GBM samples with high PBX3 expression compared with those with low PBX3 expression. (TIF 1080 kb)

Additional file 2: **Figure S2.** The effects of PBX3 overexpression or knockdown on glioma cell migration and invasion. (A) Representative images of wound-healing assays using U87 and U251 cells stably expressing LV-NC or LV-PBX3. (B) Quantification of wound-healing assays. **p < 0.01. (C) Representative images of transwell assays using U87 and U251 cells stably expressing LV-NC or LV-PBX3. (D) Quantification of transwell assays. **p < 0.01. (E) Representative images of wound-healing assays using U87 and U251 cells stably expressing LV-siRNA-NC or LV-siRNA-PBX3. (F) Quantification of wound-healing assays. **p < 0.01. (G) Representative images of transwell assays using U87 and U251 cells stably expressing LV-siRNA-NC or LV-siRNA-PBX3. (H) Quantification of transwell assays. **p < 0.01. (TIF 2002 kb)

Additional file 3: **Figure S3.** GBMs with high PBX3 expression were enriched with hallmark of TGF-β. (TIF 1138 kb)

Additional file 4: **Figure S4.** Relative expression of let-7 family members as determined by qRT-PCR in U87 and U251 cells overexpressing LV-NC or LV-PBX3. (TIF 697 kb)

Additional file 5: **Figure S5.** PBX3 promotes mesenchymal transition, migration and invasion in U251 cells is mediated by LIN28/let-7b axis. (A) PBX3 overexpression remarkably upregulated LIN28 protein levels and downregulated let-7b expression in U251 cells. (B) U251 cells stably expressing LV-PBX3 or LV-NC were transfected with siRNA-NC or siRNA-LIN28 and then immunoblotting analysis of LIN28, N-cadherin, ZEB1, Slug and CD44 were performed. (C) Quantification of wound-healing (left) and transwell (right) assays. **indicates a statistical significant difference (*p* < 0.01) between LV-NC + siRNA-NC group and LV-NC + siRNA-LIN28 group. # indicates a statistical significant difference (p < 0.01) between LV-NC + siRNA-NC group and LV-PBX3 + siRNA-NC group. $ indicates a statistical significant difference (p < 0.01) between LV-PBX3 + siRNA-NC group and LV-PBX3 + siRNA-LIN28 group. (D) U251 cells stably expressing LV-PBX3 or LV-NC were transfected with pre-miR-NC or pre-let-7b and then let-7b expression were determined by qRT-PCR. **indicates a statistical significant difference (*p* < 0.01) between LV-NC + pre-miR-NC group and LV-NC + pre-let-7b group. # indicates a statistical significant difference (*p* < 0.01) between LV-NC + pre-miR-NC group and LV-PBX3 + pre-miR-NC group. $ indicates a statistical significant difference (*p* < 0.01) between LV-PBX3 + pre-miR-NC group and LV-PBX3 + pre-let-7b group. (E) U251 cells stably expressing LV-PBX3 or LV-NC were transfected with pre-miR-NC or pre-let-7b and then immunoblotting analysis of N-cadherin, ZEB1, Slug and CD44 were performed. (F) Quantification of wound-healing (left) and transwell (right) assays. **indicates a statistical significant difference (*p* < 0.01) between LV-NC + pre-miR-NC group and LV-NC + pre-let-7b group. # indicates a statistical significant difference (*p* < 0.01) between LV-NC + pre-miR-NC group and LV-PBX3 + pre-miR-NC group. $ indicates a statistical significant difference (*p* < 0.01) between LV-PBX3 + pre-miR-NC group and LV-PBX3 + pre-let-7b group. (G) PBX3 knockdown remarkably downregulated LIN28 protein levels and upregulated let-7b expression in U251 cells. (H) U251 cells stably expressing LV-siRNA-PBX3 or LV-siRNA-NC were transfected with LIN28 overexpressing plasmids or empty vectors and then immunoblotting analysis of LIN28, N-cadherin, ZEB1, Slug and CD44 were performed. (I) Quantification of wound-healing (left) and transwell (right) assays. **indicates a statistical significant difference (*p* < 0.01) between LV-siRNA-NC + vector group and LV-siRNA-NC + LIN28 group. # indicates a statistical significant difference (p < 0.01) between LV-siRNA-NC + vector group and LV-siRNA-PBX3 + vector group. $ indicates a statistical significant difference (*p* < 0.01) between LV-siRNA-PBX3 + vector group and LV-siRNA-PBX3 + LIN28 group. (J) U251 cells stably expressing LV-siRNA-PBX3 or LV-siRNA-NC were transfected with anti-miR-NC or anti-let-7b and then let-7b expression were determined by qRT-PCR. **indicates a statistical significant difference (*p* < 0.01) between LV-siRNA-NC + anti-miR-NC group and LV-siRNA-NC + anti-let-7b group. # indicates a statistical significant difference (*p* < 0.01) between LV-siRNA-NC + anti-miR-NC group and LV-siRNA-PBX3 + anti-miR-NC group. $ indicates a statistical significant difference (*p* < 0.01) between LV-siRNA-PBX3 + anti-miR-NC group and LV-siRNA-PBX3 + anti-let-7b group. (K) U251 cells stably expressing LV-siRNA-PBX3 or LV-siRNA-NC were transfected with anti-miR-NC or anti-let-7b and then immunoblotting analysis of N-cadherin, ZEB1, Slug and CD44 were performed. (L) Quantification of wound-healing (left) and transwell (right) assays. **indicates a statistical significant difference (*p* < 0.01) between LV-siRNA-NC + anti-miR-NC group and LV-siRNA-NC + anti-let-7b group. # indicates a statistical significant difference (*p* < 0.01) between LV-siRNA-NC + anti-miR-NC group and LV-siRNA-PBX3 + anti-miR-NC group. $ indicates a statistical significant difference (*p* < 0.01) between LV-siRNA-PBX3 + anti-miR-NC group and LV-siRNA-PBX3 + anti-let-7b group. (TIF 2795 kb)

Additional file 6: **Figure S6.** Inhibition of c-myc or overexpression of c-myc reversed the PBX3 overexpression- or inhibition-induced GBM cells migration, invasion and mesenchymal transition. (A and B) Quantification of wound-healing and transwell assays. **indicates a statistical significant difference (*p* < 0.01) between LV-NC + siRNA-NC group and LV-NC + siRNA-c-myc group. # indicates a statistical significant difference (*p* < 0.01) between LV-NC + siRNA-NC group and LV-PBX3 + siRNA-NC group. $ indicates a statistical significant difference (*p* < 0.01) between LV-PBX3 + siRNA-NC group and LV-PBX3 + siRNA-c-myc group. (C) U87 and U251 cells stably expressing LV-PBX3 or LV-NC were transfected with siRNA-NC or siRNA-c-myc and then immunoblotting analysis of c-myc, N-cadherin, ZEB1, Slug and CD44 were performed. (D and E) Quantification of wound-healing and transwell assays. **indicates a statistical significant difference (*p* < 0.01) between LV-siRNA-NC + vector group and LV-siRNA-NC + c-myc group. # indicates a statistical significant difference (*p* < 0.01) between LV-siRNA-NC + vector group and LV-siRNA-PBX3 + vector group. $ indicates a statistical significant difference (*p* < 0.01) between LV-siRNA-PBX3 + vector group and LV-siRNA-PBX3 + c-myc group. (F) U87 and U251 cells stably expressing LV-siRNA-PBX3 or LV-siRNA-NC were transfected with c-myc overexpressing plasmids or empty vectors and then immunoblotting analysis of c-myc, N-cadherin, ZEB1, Slug and CD44 were performed. (TIF 1118 kb)

Additional file 7: **Figure S7.** GBMs with high PBX3 expression were enriched with hallmark of IL-6-JAK-STAT3 signaling. (TIF 1464 kb)

## Abbreviations

TGFβ: Transforming growth factor-β
NF-κB: Nuclear factor κB
ATCC: American Type Culture Collection
BALB: Female Bagg albino
ChIP: Chromatin-immunoprecipitation
DMEM: Dulbecco’s modified Eagle’s medium
GBM: Glioblastoma
HMGA2: High mobility group A2
NCBI-GEO: National Center for Biotechnology Information Gene Expression Omnibus
PBX3: Pre-B-cell leukemia homebox 3
PMA: Phorbol 12-myristate 13-acetate
PMT: Proneural-to-mesenchymal
qRT-PCR: Quantitative real-time polymerase chain reaction
Rembrandt: Repository for Molecular Brain Neoplasia Data
SMAD: Small mother against decapentaplegic
SNAI1: Snail family transcriptional repressor 1
STAT3: Signal transducer and activator of transcription 3
WT: Wild-type
