# Supplementary figures and images for: PBX3/MEK/ERK1/2/LIN28/let-7b positive feedback loop enhances mesenchymal phenotype to promote glioblastoma migration and invasion
Source: J Exp Clin Cancer Res. 2018 Jul 17;37:158. doi: 10.1186/s13046-018-0841-0 (PMC6050701; doi:10.1186/s13046-018-0841-0)

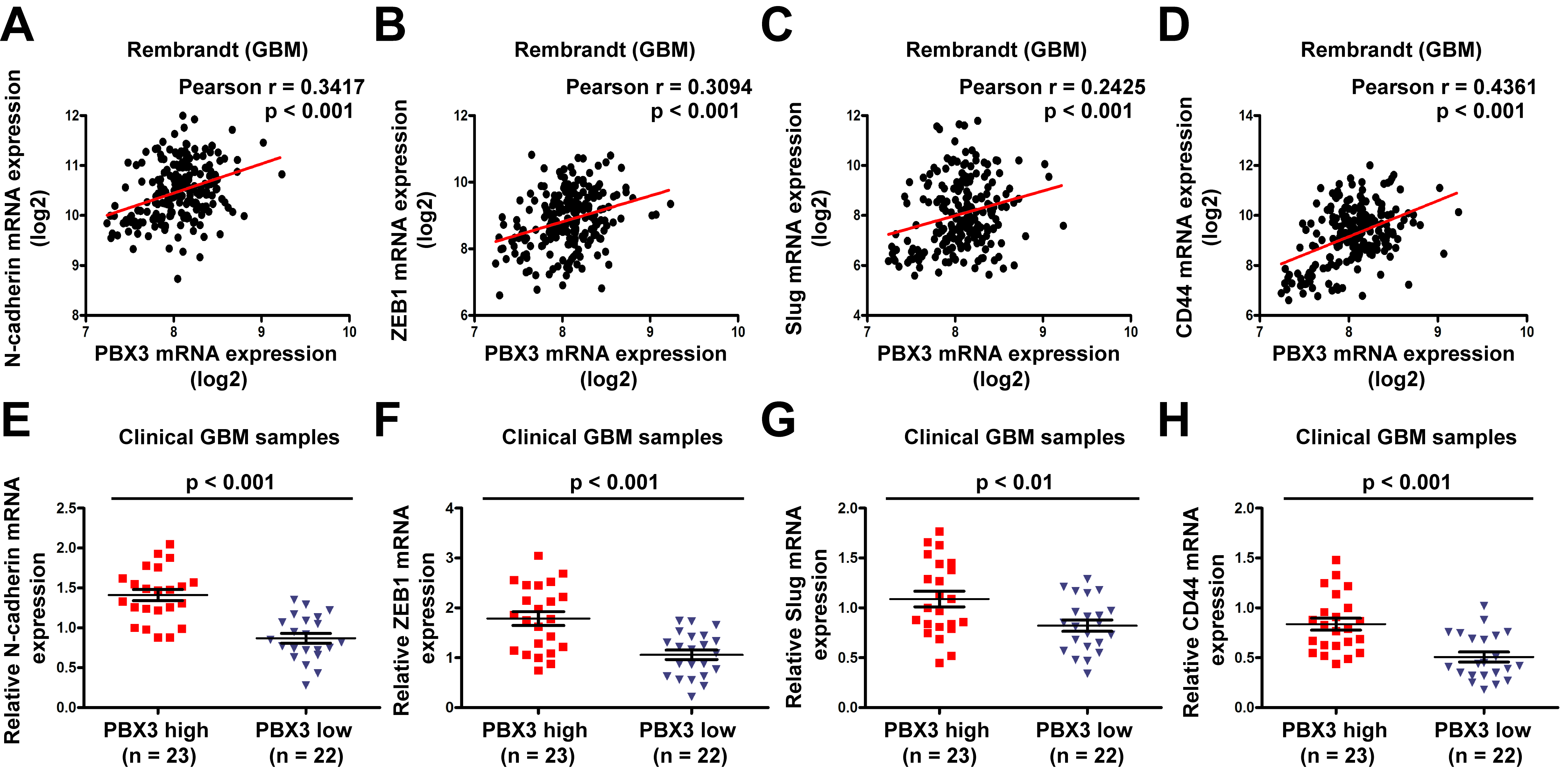

Supplement: Supplementary file 1 — Figure S1. PBX3 is positively correlated with mesenchymal markers. (A-D) Pearson’s correlation analyses indicate that PBX3 expression is positively associated with N-cadherin (A), ZEB1 (B), Slug (C), and CD44 (D) expressions in Rembrandt database. (E-H) The expression of N-cadherin (E), ZEB1 (F), Slug (G), and CD44 (H) are increased in GBM samples with high PBX3 expression compared with those with low PBX3 expression. (TIF 1080 kb) [file 13046_2018_841_MOESM1_ESM.tif]

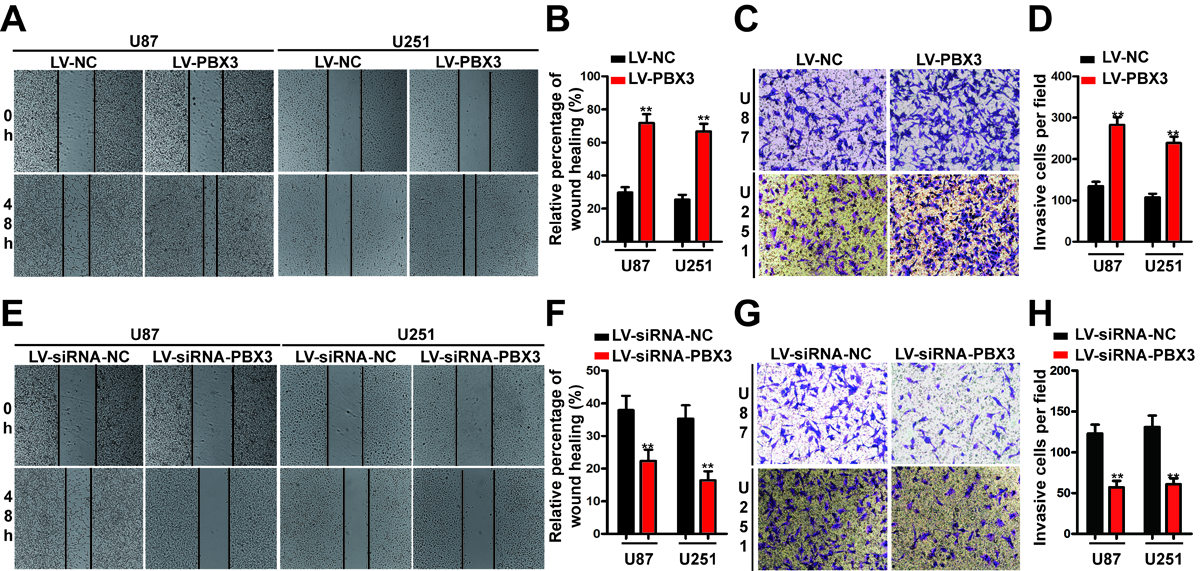

Supplement: Supplementary file 2 — Figure S2. The effects of PBX3 overexpression or knockdown on glioma cell migration and invasion. (A) Representative images of wound-healing assays using U87 and U251 cells stably expressing LV-NC or LV-PBX3. (B) Quantification of wound-healing assays. **p < 0.01. (C) Representative images of transwell assays using U87 and U251 cells stably expressing LV-NC or LV-PBX3. (D) Quantification of transwell assays. **p < 0.01. (E) Representative images of wound-healing assays using U87 and U251 cells stably expressing LV-siRNA-NC or LV-siRNA-PBX3. (F) Quantification of wound-healing assays. **p < 0.01. (G) Representative images of transwell assays using U87 and U251 cells stably expressing LV-siRNA-NC or LV-siRNA-PBX3. (H) Quantification of transwell assays. **p < 0.01. (TIF 2002 kb) [file 13046_2018_841_MOESM2_ESM.tif]

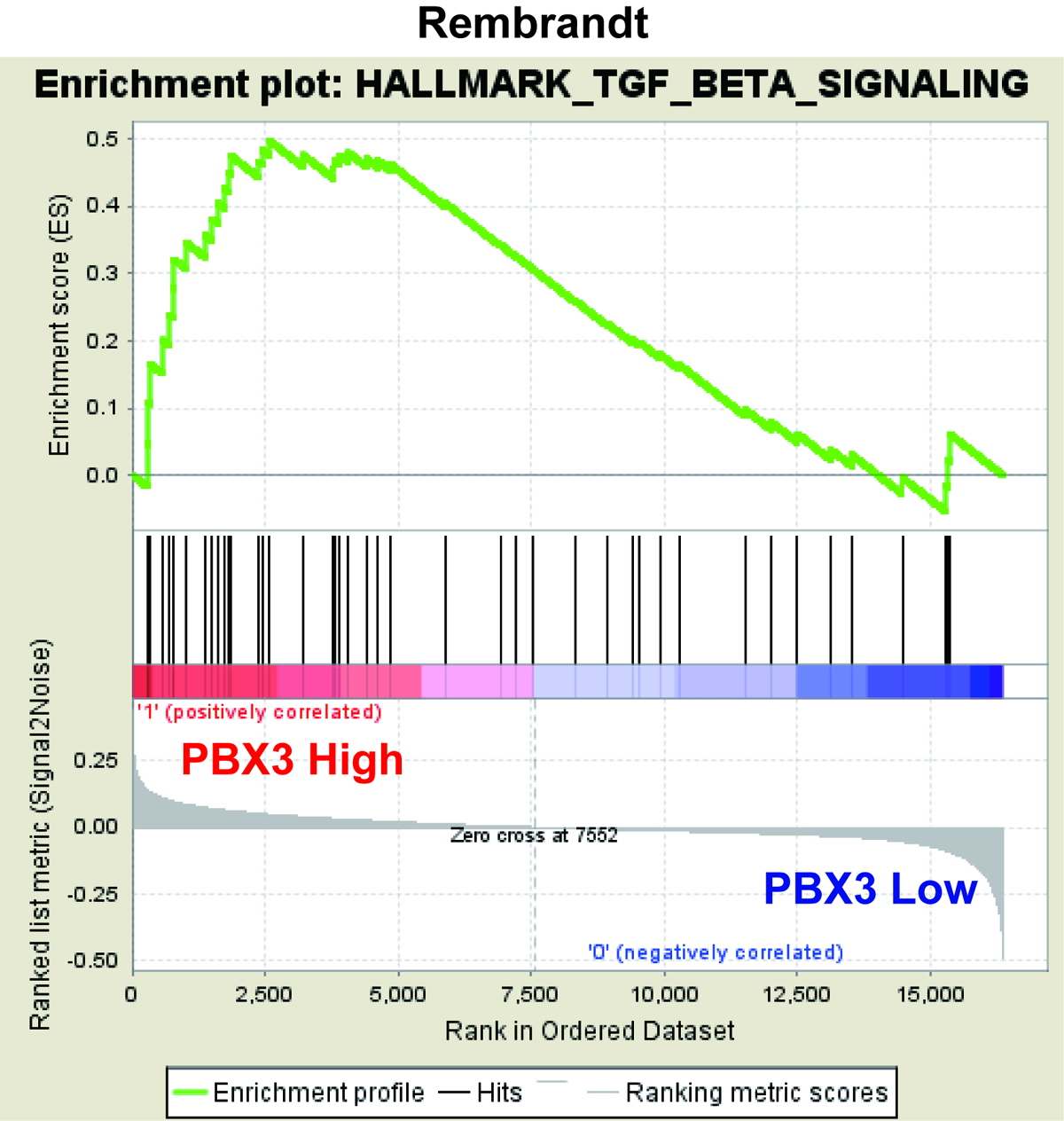

Supplement: Supplementary file 3 — Figure S3. GBMs with high PBX3 expression were enriched with hallmark of TGF-β. (TIF 1138 kb) [file 13046_2018_841_MOESM3_ESM.tif]

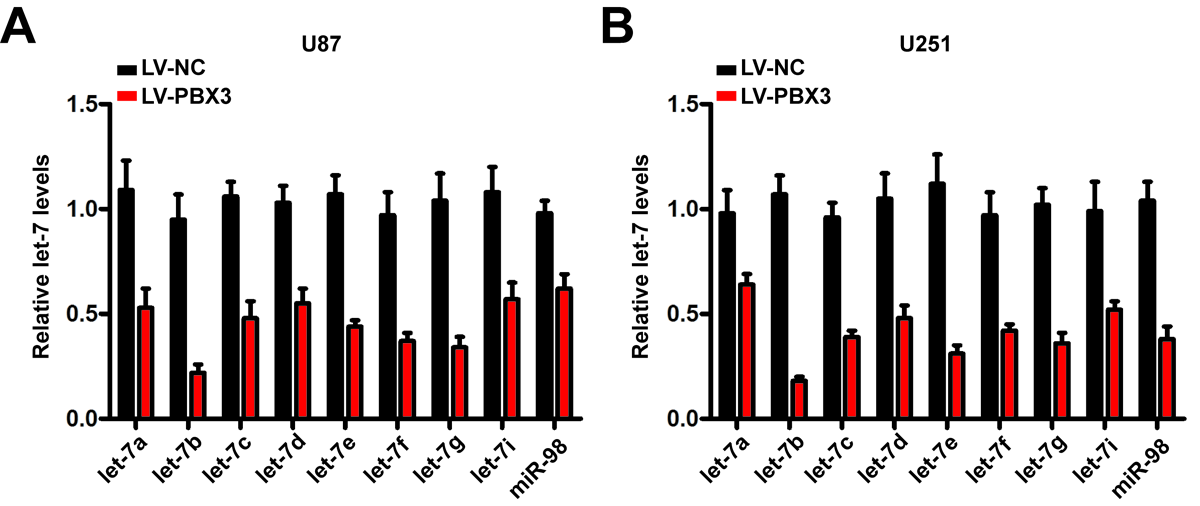

Supplement: Supplementary file 4 — Figure S4. Relative expression of let-7 family members as determined by qRT-PCR in U87 and U251 cells overexpressing LV-NC or LV-PBX3. (TIF 697 kb) [file 13046_2018_841_MOESM4_ESM.tif]

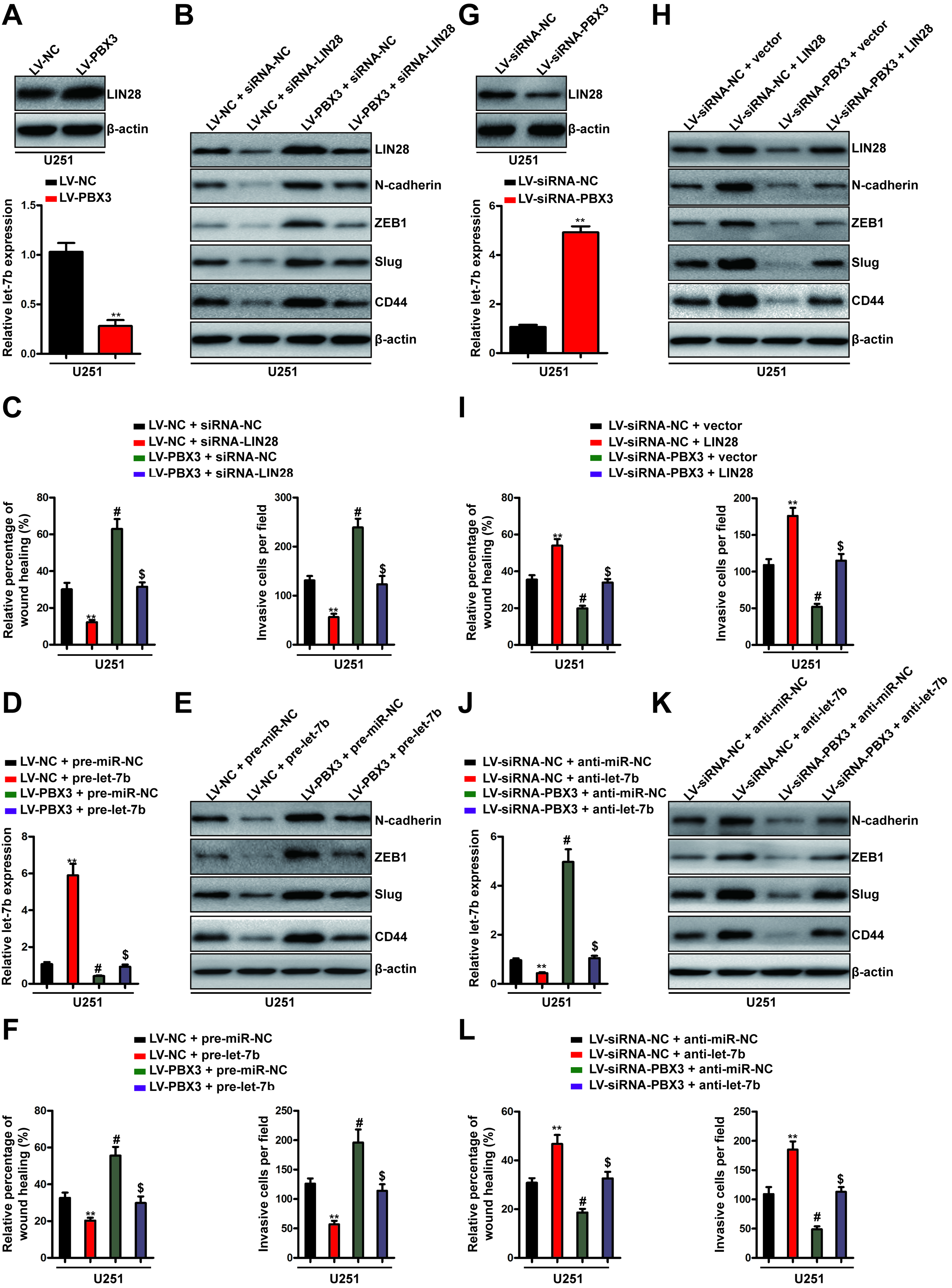

Supplement: Supplementary file 5 — Figure S5. PBX3 promotes mesenchymal transition, migration and invasion in U251 cells is mediated by LIN28/let-7b axis. (A) PBX3 overexpression remarkably upregulated LIN28 protein levels and downregulated let-7b expression in U251 cells. (B) U251 cells stably expressing LV-PBX3 or LV-NC were transfected with siRNA-NC or siRNA-LIN28 and then immunoblotting analysis of LIN28, N-cadherin, ZEB1, Slug and CD44 were performed. (C) Quantification of wound-healing (left) and transwell (right) assays. **indicates a statistical significant difference (p < 0.01) between LV-NC + siRNA-NC group and LV-NC + siRNA-LIN28 group. # indicates a statistical significant difference (p < 0.01) between LV-NC + siRNA-NC group and LV-PBX3 + siRNA-NC group. $ indicates a statistical significant difference (p < 0.01) between LV-PBX3 + siRNA-NC group and LV-PBX3 + siRNA-LIN28 group. (D) U251 cells stably expressing LV-PBX3 or LV-NC were transfected with pre-miR-NC or pre-let-7b and then let-7b expression were determined by qRT-PCR. **indicates a statistical significant difference (p < 0.01) between LV-NC + pre-miR-NC group and LV-NC + pre-let-7b group. # indicates a statistical significant difference (p < 0.01) between LV-NC + pre-miR-NC group and LV-PBX3 + pre-miR-NC group. $ indicates a statistical significant difference (p < 0.01) between LV-PBX3 + pre-miR-NC group and LV-PBX3 + pre-let-7b group. (E) U251 cells stably expressing LV-PBX3 or LV-NC were transfected with pre-miR-NC or pre-let-7b and then immunoblotting analysis of N-cadherin, ZEB1, Slug and CD44 were performed. (F) Quantification of wound-healing (left) and transwell (right) assays. **indicates a statistical significant difference (p < 0.01) between LV-NC + pre-miR-NC group and LV-NC + pre-let-7b group. # indicates a statistical significant difference (p < 0.01) between LV-NC + pre-miR-NC group and LV-PBX3 + pre-miR-NC group. $ indicates a statistical significant difference (p < 0.01) between LV-PBX3 + pre-miR-NC group and LV-P [file 13046_2018_841_MOESM5_ESM.tif]

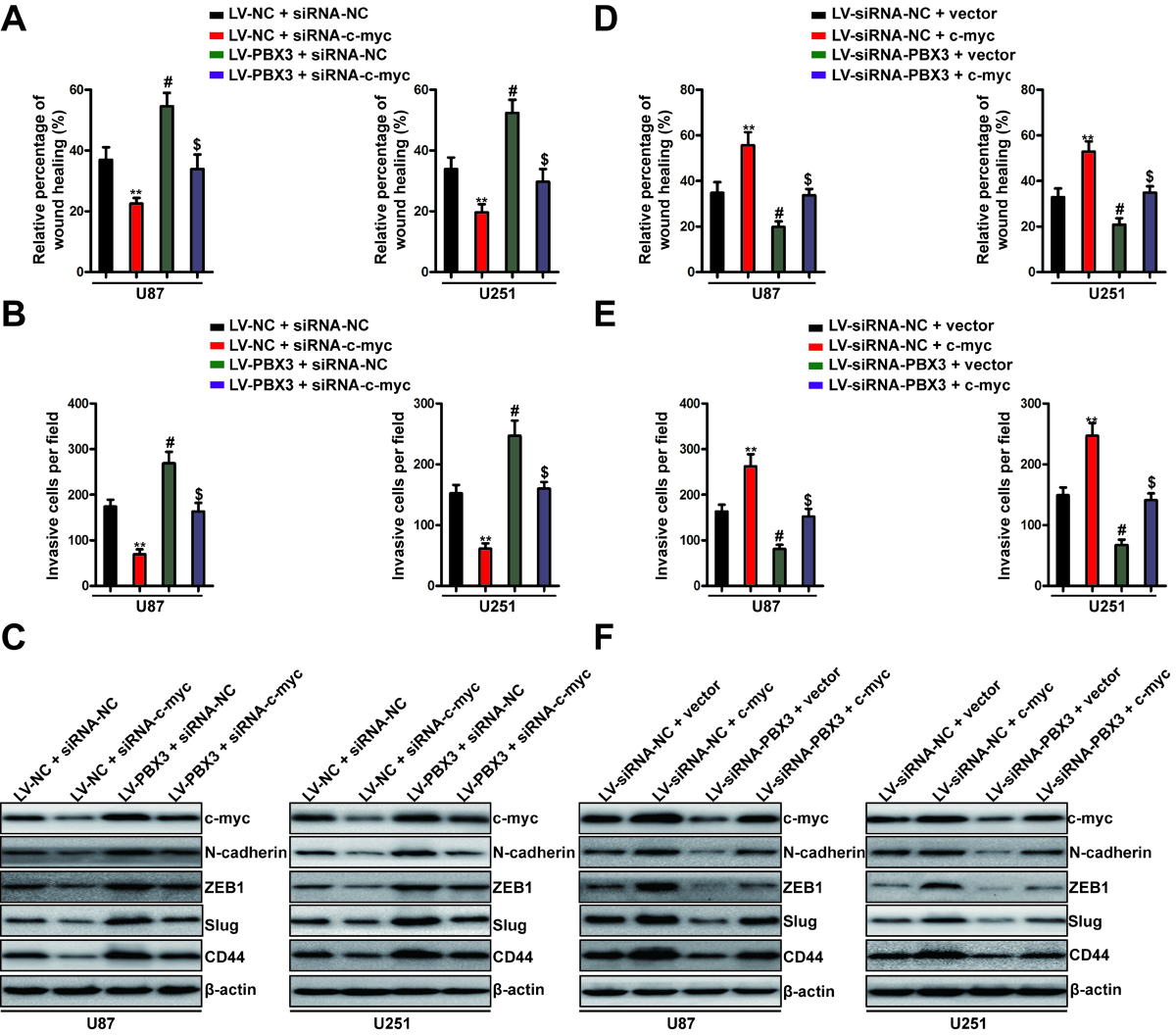

Supplement: Supplementary file 6 — Figure S6. Inhibition of c-myc or overexpression of c-myc reversed the PBX3 overexpression- or inhibition-induced GBM cells migration, invasion and mesenchymal transition. (A and B) Quantification of wound-healing and transwell assays. **indicates a statistical significant difference (p < 0.01) between LV-NC + siRNA-NC group and LV-NC + siRNA-c-myc group. # indicates a statistical significant difference (p < 0.01) between LV-NC + siRNA-NC group and LV-PBX3 + siRNA-NC group. $ indicates a statistical significant difference (p < 0.01) between LV-PBX3 + siRNA-NC group and LV-PBX3 + siRNA-c-myc group. (C) U87 and U251 cells stably expressing LV-PBX3 or LV-NC were transfected with siRNA-NC or siRNA-c-myc and then immunoblotting analysis of c-myc, N-cadherin, ZEB1, Slug and CD44 were performed. (D and E) Quantification of wound-healing and transwell assays. **indicates a statistical significant difference (p < 0.01) between LV-siRNA-NC + vector group and LV-siRNA-NC + c-myc group. # indicates a statistical significant difference (p < 0.01) between LV-siRNA-NC + vector group and LV-siRNA-PBX3 + vector group. $ indicates a statistical significant difference (p < 0.01) between LV-siRNA-PBX3 + vector group and LV-siRNA-PBX3 + c-myc group. (F) U87 and U251 cells stably expressing LV-siRNA-PBX3 or LV-siRNA-NC were transfected with c-myc overexpressing plasmids or empty vectors and then immunoblotting analysis of c-myc, N-cadherin, ZEB1, Slug and CD44 were performed. (TIF 1118 kb) [file 13046_2018_841_MOESM6_ESM.tif]

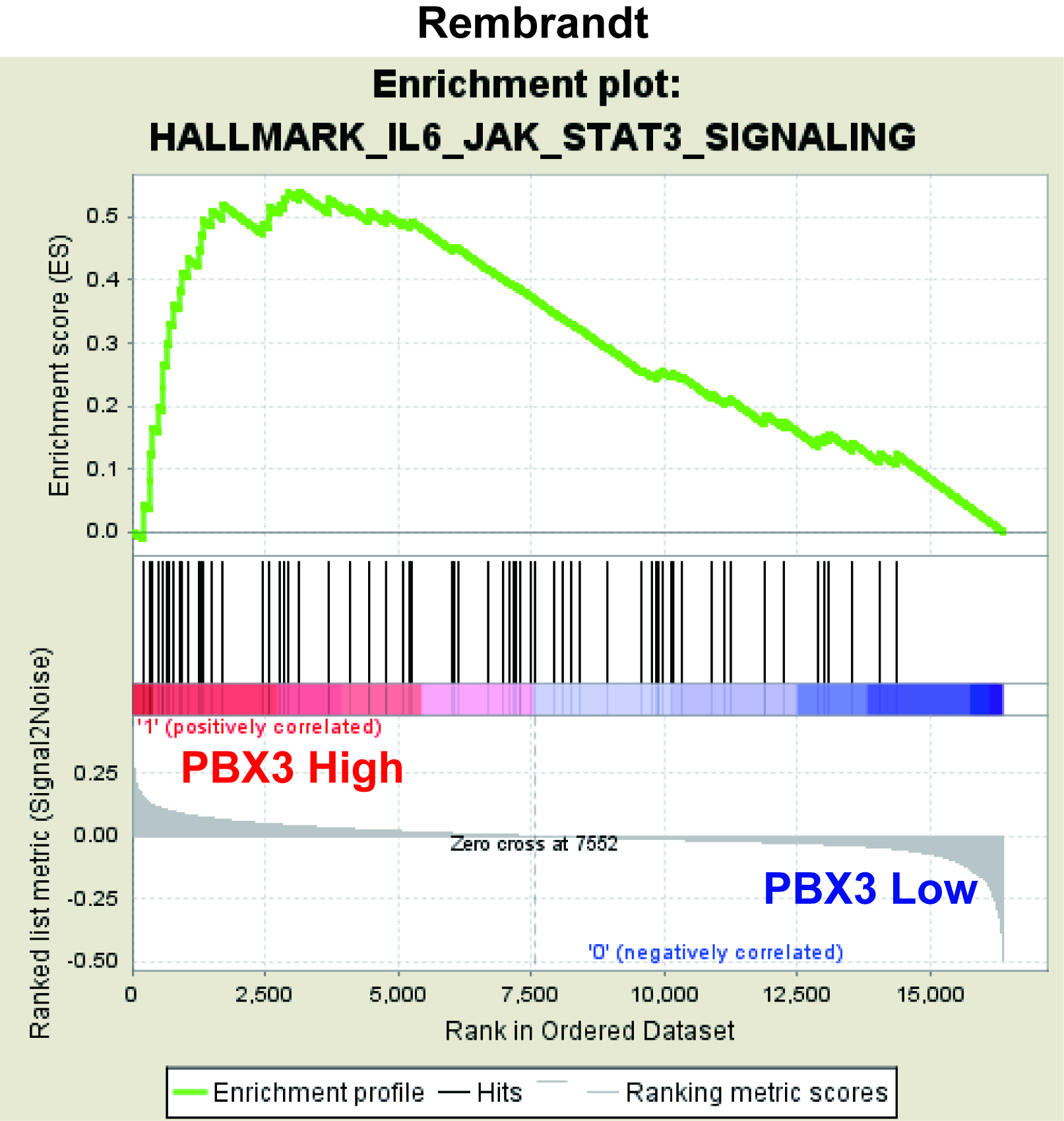

Supplement: Supplementary file 7 — Figure S7. GBMs with high PBX3 expression were enriched with hallmark of IL-6-JAK-STAT3 signaling. (TIF 1464 kb) [file 13046_2018_841_MOESM7_ESM.tif]
